# Changing soil carbon: influencing factors, sequestration strategy and research direction

**DOI:** 10.1186/s13021-020-0137-5

**Published:** 2020-02-17

**Authors:** Shangqi Xu, Chunlei Sheng, Chunjie Tian

**Affiliations:** Key Laboratory of Mollisols Agroecology, Northeast Institute of Geography and Agroecology, Chinese Academy of Sciences, 4888 Shengbei Road, Changchun, 130102 China

**Keywords:** Soil carbon, Meta-analysis, Climate change, Human activities, Land use change, Agricultural management, Wetland, Carbon budgets

## Abstract

Soil carbon (C) plays a critical role in the global C cycle and has a profound effect on climate change. To obtain an in-depth and comprehensive understanding of global soil C changes and better manage soil C, all meta-analysis results published during 2001–2019 relative to soil C were collected and synthesized. The effects of 33 influencing factors on soil C were analyzed, compared and classified into 5 grades according to their effects on soil C. The effects of different categories of influencing factors, including land use change (LUC), management and climate change, on soil C and the underlying mechanism were compared and discussed. We propose that natural ecosystems have the capacity to buffer soil C changes and that increasing C inputs is one of the best measures to sequester C. Furthermore, a comparison between the meta-analyses and previous studies related to soil C based on bibliometric analysis suggested that studies on wetland soil C, soil C budgets and the effects of pollution and pesticides on soil C should be strengthened in future research.

## Background

Soil is the largest terrestrial ecosystem C pool, at approximately 2500 Pg C, and this pool is 3.3 times the size of the atmospheric C pool (760 Pg) [1, 2]. Soil has great potential for mitigating C emissions, and the C emissions from soil can be reduced to 50% by 2050 of those in 2010 with suitable mitigation practices [3]. Determining how to explore the C sink function of soil with suitable management practices is very important for global change mitigation. However, soil is a very complex system, and the soil C pool is influenced by multiple factors, including climate change, soil management, land use change (LUC), and so on [4, 5]. A synthesis and comprehensive analysis of the influence of different factors on soil C can provide support for soil C management and climatic change mitigation. There have been many achievements in soil C research, but most previous studies have focused on a few factors or a few ecosystems. A comprehensive global analysis of soil C under various influencing factors is still lacking and may impede an in-depth understanding of the global soil C cycle and soil C management.

**Fig. 1:**
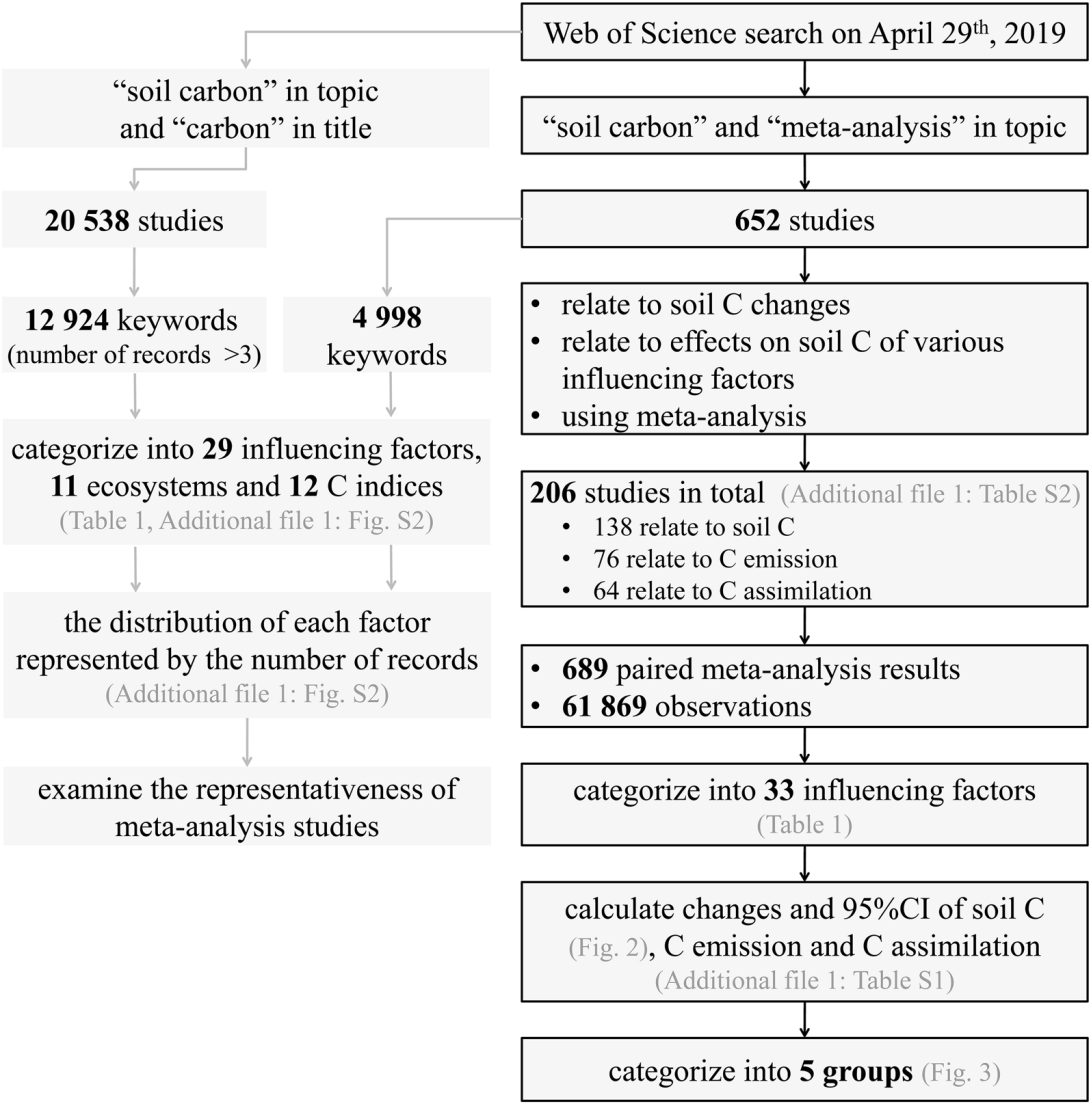
Flow chart of this study. The keywords included Keywords-Author, Keywords-Plus, and Phrases-Title, which were obtained using Thomson Data Analyzer

A meta-analysis is the most powerful method for synthesizing the results of different studies conducted under various conditions to evaluate the direction, magnitude and response patterns due to the effects of influencing factors [6], and these analyses on soil C have been conducted increasingly in recent years (Additional file 1: Fig. S1). Here, studies on soil C using a meta-analysis from 2001 to 2019 (Web of Science search on April 29th, 652 studies) were collected, and the results of the meta-analysis were analyzed and synthesized comprehensively. Simultaneously, all studies related to soil C (20 538 studies) were collected, and a bibliometric analysis was conducted using Thomson Data Analyzer software (v6.0, Thomson Reuters, New York, USA) to analyze and compare the keyword distribution of these studies and the collected meta-analysis studies (Fig. 1). Our objectives were (1) to understand the current research overview of global studies on soil C; (2) to understand C changes due to the effects of different factors in various ecosystems and provide insight into global soil C changes; and (3) to provide theoretical support for soil C management with the aim of C sequestration and global change mitigation.

## Main text

### Effects on soil C of different influencing factors

**Fig. 2:**
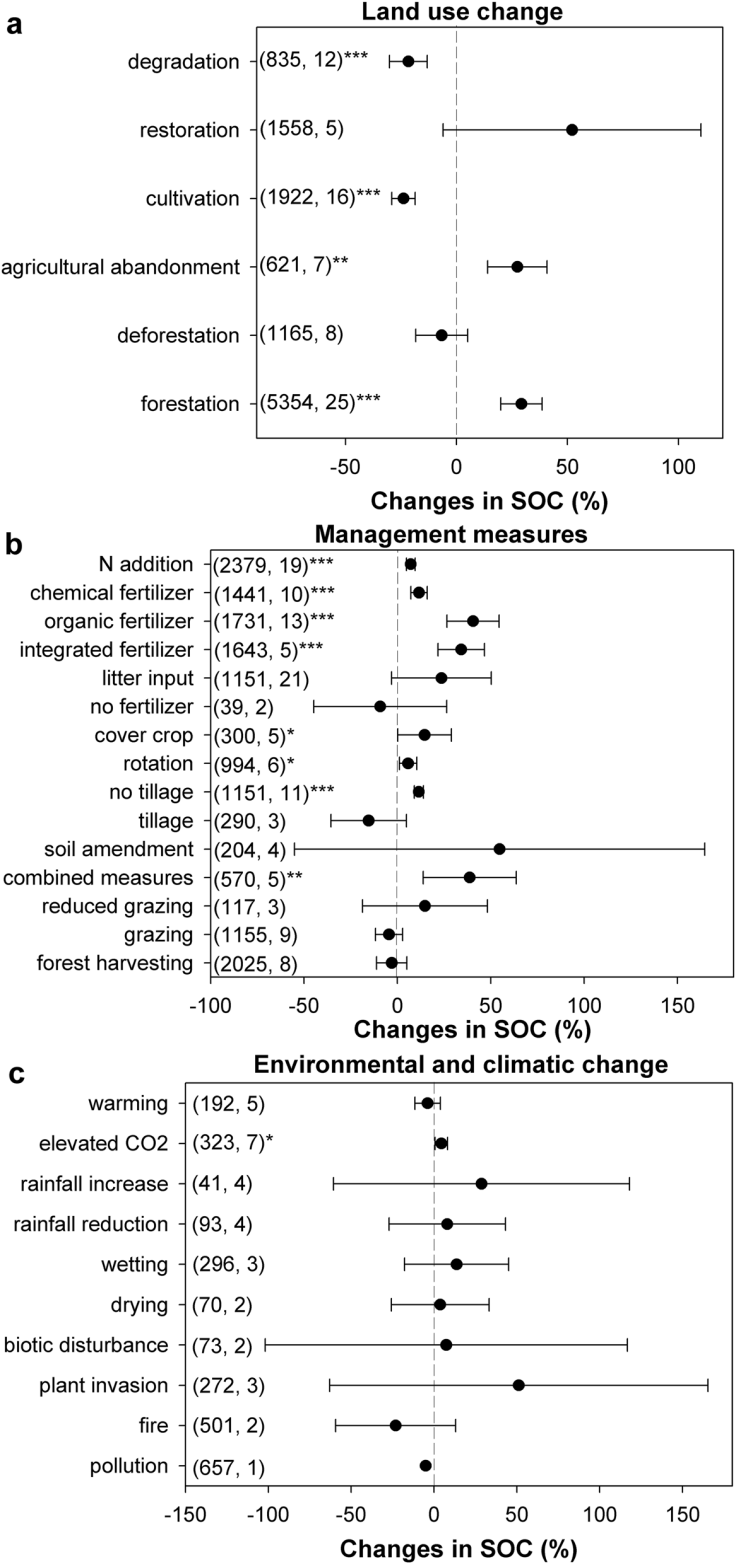
Changes in soil C due to the effects of each factor. The circles and error bars represent the means and 95% confidence intervals; the numbers next to the Y axes indicate the number of observations and studies. The results are significant at *p *< 0.05 (*), *p *< 0.01 (**), and *p *< 0.001 (***)

**Fig. 3:**
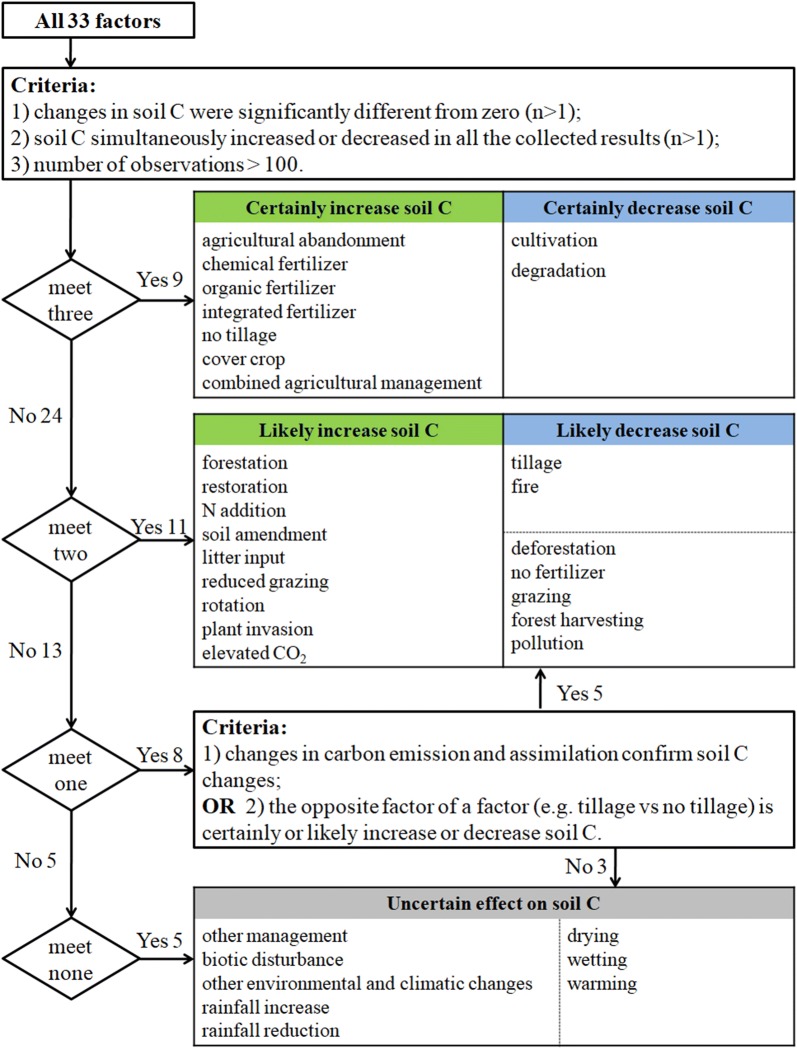
Flow chart of the classification of each influencing factor. The changes in soil C were examined with a single sample t-test, and a significance level of 0.05 was used. Furthermore, “n” is the number of collected meta-analysis results. The numbers followed by “Yes” or “No” are the number of factors that met or did not meet the classification criteria

In total, the results of 61 869 observations within 689 paired experiments were collected from 206 studies that were related to the meta-analysis on soil C changes under the effects of different factors (Fig. 1). All data were collected either directly from tables and/or text or from figures using GetData Graph Digitizer software (v2.22). Finally, 33 influencing factors that were collected from the studies fell into three categories: LUC, management and climatic change. The changes in soil C due to the effects of each factor are shown in Fig. 2. According to the confidence of their influence on soil C change, these 33 factors were classified into five groups, including certainly increase soil C (7 factors), certainly decrease soil C (2 factors), likely increase soil C (9 factors), likely decrease soil C (7 factors), and uncertain effect on soil C (8 factors) (Fig. 3).

All LUC factors had a clear effect (certainly/likely increase/decrease soil C) on soil C, which indicated that LUC factors always lead to significant changes in soil C (Fig. 2a). The change from farmland to other ecosystem types (e.g., agricultural abandonment) always led to a soil C increase, and soil C always decreased when other ecosystem types changed to farmland (e.g., cultivation); the condition for forests was opposite that for farmland. These results indicated that substantial potential C sinks exist in farmland area and that farmland reforestation is the most suggested practice for climatic change mitigation [7–9]. Furthermore, previous studies indicated that there was less soil C in farmland than in other ecosystems, while there was more soil C in forests than in other ecosystems [10, 11]. Thus, it is inferred that under the influence of the same factors, soil with a relatively lower C content has more potential to increase its C content than soil with a relatively higher C content; however, soil with a relatively higher C content is more likely to decrease its C content than soil with a relatively lower C content. This phenomenon was observed in previous studies [4, 12], suggesting that soil may have the capacity to buffer soil C changes. Specifically, in certain environments, the soil has the capacity to maintain the C content within a relatively stable range; when the soil C content is higher or lower than this range, the C content tends to return to this range.

Regarding different management types, 11 measures out of 16 increased the soil C content, with 8 measures significantly increasing the soil C content (Fig. 2b). For the 13 factors related to farmland, except tillage and no fertilizer, all of the other 11 management practices related to farmland increased the soil C content. These results indicated that there are many opportunities to increase the soil C content via reasonable management, especially in farmland [7]. The results showed that almost all the management benefits to plant biomass led to soil C increases, e.g., all fertilizer management, forestation, restoration, soil amendments, and litter inputs. Reducing plant biomass always led to a decrease in soil C, e.g., deforestation, fire, grazing, forest harvesting and no fertilization. Tillage was an exception because compared to no tillage, the soil C decreased, although the crop biomass increased. This result was because tillage increased the soil C decomposition rate significantly [13], and the increase in crop biomass was less than the soil C losses [14]. According to these results, the effect of the factors on soil C was dependent on changes in C inputs and C decomposition. In other words, the soil C content was determined by the relationship between C inputs and C decomposition [15, 16]. However, the relationship has rarely been discussed, and most previous studies relative to soil C have focused only on C inputs or soil C decomposition. The C decomposition rate was dependent on microbial activities, which were mostly influenced by the environment and substrates [15]. Thus, the C decomposition rate was fixed in a certain environment. It can be concluded that in certain environments, the soil C stocks were mainly dependent on C inputs. Regarding the different influencing factors, changes in C inputs were more influential on the soil C content than changes in C decomposition [17, 18]. Soil C stocks may show an exponential increase with increasing C inputs while showing a reciprocal decrease with increasing C decomposition. Thus, to sequester C, the most practical measure is to increase C inputs and avoid an increase in the C decomposition rate. However, we should first guarantee that the C inputs are higher than the C decomposition rate.

All of the climate change factors had uncertain effects on the soil C content, except elevated CO_2_, which was identified as likely increasing the soil C content (Fig. 2c). This result was because most of the climate change factors influenced plant growth and soil C decomposition simultaneously, e.g., warming increased plant biomass, but the C decomposition rate also increased; thus, the final effects on soil C were ambiguous [19]. However, elevated CO_2_ was beneficial for plant growth but had a weak effect on C decomposition [20–22]. The effects of elevated CO_2_ on soil C indicated that the natural ecosystem had a capacity to buffer elevated CO_2_ that was similar to the capacity to buffer soil C. However, due to the degradation of natural ecosystems and wide, sharp changes in CO_2_ and soil C, this buffer capacity dwindled significantly. Thus, to mitigate climatic change, it is still necessary to take appropriate measures to sequester C. Moreover, in soil C management, we should take advantage of this buffer capacity of natural ecosystems. For instance, to sequester soil C, soils that have experienced sharp C losses or have relatively lower C contents should be prioritized.

It should be noted that some studies were included in different meta-analyses, and these studies may give more weight to the results than other studies. Although this may reduce the accuracy of the results, the influence was very limited because the results were aggregated from very extensive studies and each meta-analysis had weighted each included study. Therefore, the results of this study are still reliable and more importantly, they are very valuable.

### Fields should be strengthened in future research

In the bibliometric analysis, all keywords were classified into 52 groups, including 29 influencing factors, 11 ecosystems and 12 C indices (Table 1, Additional file 1: Fig. S2). The results showed that the studies related to soil C were highly centralized in farmland, forest, and grassland ecosystems, while other ecosystems, including wetland, desert, tundra, and barren land ecosystems, received very little attention (Additional file 1: Fig. S2). Regarding the different C indices, most studies focused on soil organic carbon (SOC), CO_2_, C fractions, and C sequestration, while few studies focused on CH_4_, ecosystem C, C budgets, and C stocks. Regarding the different influencing factors, much attention has focused on climatic change, tillage, LUC, and litter and straw, while little attention has been placed on rainfall, invasion, pesticides, and soil amendments.

**Table 1:** Classification standard of each influencing factor in the collected results of the meta-analysis and its corresponding keyword classification in the bibliometric analysis

Groups	Influencing factor	Standard of classification	Corresponding keyword classification
Land use change (LUC)	Degradation	Ecosystem degradation, including diversity loss and loss of other ecological function	Degradation
	Restoration	The restoration of degraded or cultivated ecosystems, including vegetation recovery	Restoration
	Cultivation	Agricultural cultivation on other ecosystems	Cultivation
	Forestation	Forestation of other ecosystems, including plantation and agroforestry	Afforestation
	Agricultural abandonment	Changes from farmland to other ecosystems except forest, as farmland to forest was included in forestation	LUC
	Deforestation	Changes from forest to other ecosystems except farmland, as forest to farmland was included in cultivation	LUC
Management	N addition	Including N fertilization, N addition and N deposition	N management
	Chemical fertilizer	Other chemical fertilizer including N or without N	Chemical fertilizer
	Organic fertilizer	Organic fertilizer, organic manure, organic amendments and so on	Organic fertilizer
	Integrated fertilizer	Fertilization including both chemical fertilizer and organic fertilizer	Human activities
	Litter input	Including straw return, litter input and other organic matter inputs	Litter or straw
	No fertilizer	No fertilizer or organic matter input	Other factors
	Cover crop	Cover crop	Litter or straw
	Rotation	Cropping system including different crops or intercropping	Rotation
	No tillage	Including reduced tillage and zero tillage	Tillage
	Tillage	Including plow tillage, rotation tillage, deep tillage and others	Tillage
	Soil amendment	Soil amending with amendment inputs, including biochar, gypsum, lime, and others	Biochar, soil amendment
	Combined agricultural management	Management with at least 2 different agricultural measures, including organic farming and conservational farming	Agricultural management
	Reduced grazing	Including grazing exclusion and reduction	Grazing
	Grazing	Including different intensities and frequencies of grazing	Grazing
	Forest harvesting	Including whole tree harvest, stem harvesting, partial harvesting and different intensive harvesting	Forest management
	Other management	Film mulching, new rice varieties, inhibitors and so on	Agricultural management, pesticides
Environmental and climatic change	Warming	Including temperature increases, experimental warming and others	Warming
	Elevated CO_2_	Elevated CO_2_	Elevated CO_2_
	Rainfall increase	Rainfall increase	Rainfall
	Rainfall reduction	Rainfall reduction	Rainfall
	Wetting	Rewetting of drainage ecosystems or irrigation	Drainage or wetting
	Drying	Including the drainage of flooded areas and drought in uplands	Drainage or wetting
	Biotic disturbance	Soil disturbance by soil fauna, such as earthworms and so on	Biotic disturbance
	Plant invasion	Exotic plants invade the original ecosystem	Invasions
	Fire	Including wildfire and fire management	Fire
	Pollution	Including metal pollution, waste pollution, organic contamination, acid rain, and so on	Pollution
	Other environmental and climatic changes	Including increased snowpack, elevated UV-B, attenuated UV-B, elevated O_3_, freeze–thaw and so on	Climate change, environmental gradient

The categorization of keywords in the bibliometric analysis (29 influencing factors) and the influencing factors in the collected results of the meta-analysis (33 influencing factors) were not exactly the same because the information obtained via the two methods was different

The keywords of the meta-analysis studies and all studies related to soil C had similar distributions, which indicated that the results from the meta-analysis studies could well represent all studies related to soil C (Additional file 1: Fig. S2). The results of in-depth comparative analyses also found some research fields in which relatively few meta-analyses have been conducted, such as wetlands, C fractions, C budgets, pesticides, cultivation, degradation and tillage. To better understand global soil C changes, further meta-analyses should be conducted in these fields. Notably, few studies have focused on wetlands and C budgets, not only in meta-analysis studies but also in all studies related to soil C. However, wetlands and C budgets are important aspects of this topic.

Wetlands are the most C-rich ecosystems on Earth, accounting for only 5–8% of the global land area but representing 20–30% or more of the global C stock [23]. Furthermore, wetlands are one of the most vulnerable and seriously threatened ecosystems on Earth. Approximately half of global wetlands have been lost or degraded due to human disturbance, and this loss has profound impacts on the global C cycle [24, 25].

C budgets are the best way to estimate soil C changes due to the effects of various influencing factors, and these calculations can improve the accuracy of global C estimations [15, 26]. However, the calculation of C budges is complicated, and no uniform calculation standard has been approved; as a result, few studies have been conducted [27, 28]. Thus, a relatively simpler C budget model may be a better choice to accurately estimate soil C. As mentioned above, soil C depends on C inputs and decomposition. Thus, a simple model based on these two aspects may be meaningful.

## Conclusions

In conclusion, forestation is an effective method to sequester C, especially in farmland ecosystems. However, in most conditions, extensive forestation or agricultural abandonment is unrealistic due to policy and cost reasons. Instead, conserving natural ecosystems and restoring degraded ecosystems are feasible and effective methods to sequester C and benefit ecological protection. Second, suitable soil management can explore the huge soil C sink potential, especially for soils with relatively lower C contents, such as farmland soils. Management techniques that can increase C inputs, such as organic fertilizer applications, litter inputs and organic agriculture, are strongly recommended because of their high efficiency in accelerating soil C sequestration and their benefit to the sustainable development of agriculture. In other words, the two most important aspects of C sequestration are avoiding increases in the C decomposition rate and facilitating C inputs as much as possible. Furthermore, it is essential to conduct more studies on aspects that have profound impacts on global soil C change but receive little attention, including changes in wetland soil C, soil C budgets and the effects of pollution and pesticides on soil C.

## Supplementary information

**Additional file 1: Fig. S1.** The numbers of published papers with meta-analyses related to soil carbon and all studies related to soil carbon from 2009 to 2018. **Fig. S2.** Keyword distributions of meta-analyses and all studies related to soil carbon. The bar plot indicates the percentage changes in keyword distributions in the 11 ecosystems, 12 indices and 29 influencing factors between the meta-analyses and all studies related to soil carbon. The network indicates the keyword distributions in the 11 ecosystems, 12 indices and 29 influencing factors and the co-occurrence frequency of two groups; the vertex indicates the relative percentage of each group; the numerical value of the vertex of each group = 50 * keyword count of each group/the maximum keyword count; and the edges indicate the co-occurrence frequency of two groups. The numerical value of the edge of each group = 50 * co-occurrence frequency of each pair of two groups/the maximum co-occurrence frequency. **Table S1.** Changes in C losses and C assimilation due to different influencing factors. The C losses include CO_2_ emissions, CH_4_ emissions and carbon decomposition. C assimilation includes underground biomass, aboveground biomass, plant biomass, net primary production and so on. NA means that no data were reported in the studies or that there were not enough data for the t-tests. **Table S2.** List of the references that the meta-analysis results were collected.

## Data Availability

The dataset supporting the conclusions of this article is included within the article and its additional file.

## Abbreviations

C: carbon
N: nitrogen
LUC: land use change
SOC: soil organic carbon
