# Supplementary material for: Changing soil carbon: influencing factors, sequestration strategy and research direction
Source: Carbon Balance Manag. 2020 Feb 17;15:2. doi: 10.1186/s13021-020-0137-5 (PMC7227295; doi:10.1186/s13021-020-0137-5)
Supplement: Supplementary file 1 — Additional file 1: Fig. S1. The numbers of published papers with meta-analyses related to soil carbon and all studies related to soil carbon from 2009 to 2018. Fig. S2. Keyword distributions of meta-analyses and all studies related to soil carbon. The bar plot indicates the percentage changes in keyword distributions in the 11 ecosystems, 12 indices and 29 influencing factors between the meta-analyses and all studies related to soil carbon. The network indicates the keyword distributions in the 11 ecosystems, 12 indices and 29 influencing factors and the co-occurrence frequency of two groups; the vertex indicates the relative percentage of each group; the numerical value of the vertex of each group = 50 * keyword count of each group/the maximum keyword count; and the edges indicate the co-occurrence frequency of two groups. The numerical value of the edge of each group = 50 * co-occurrence frequency of each pair of two groups/the maximum co-occurrence frequency. Table S1. Changes in C losses and C assimilation due to different influencing factors. The C losses include CO2 emissions, CH4 emissions and carbon decomposition. C assimilation includes underground biomass, aboveground biomass, plant biomass, net primary production and so on. NA means that no data were reported in the studies or that there were not enough data for the t-tests. Table S2. List of the references that the meta-analysis results were collected. [file 13021_2020_137_MOESM1_ESM.pdf]

**Additional file 1**

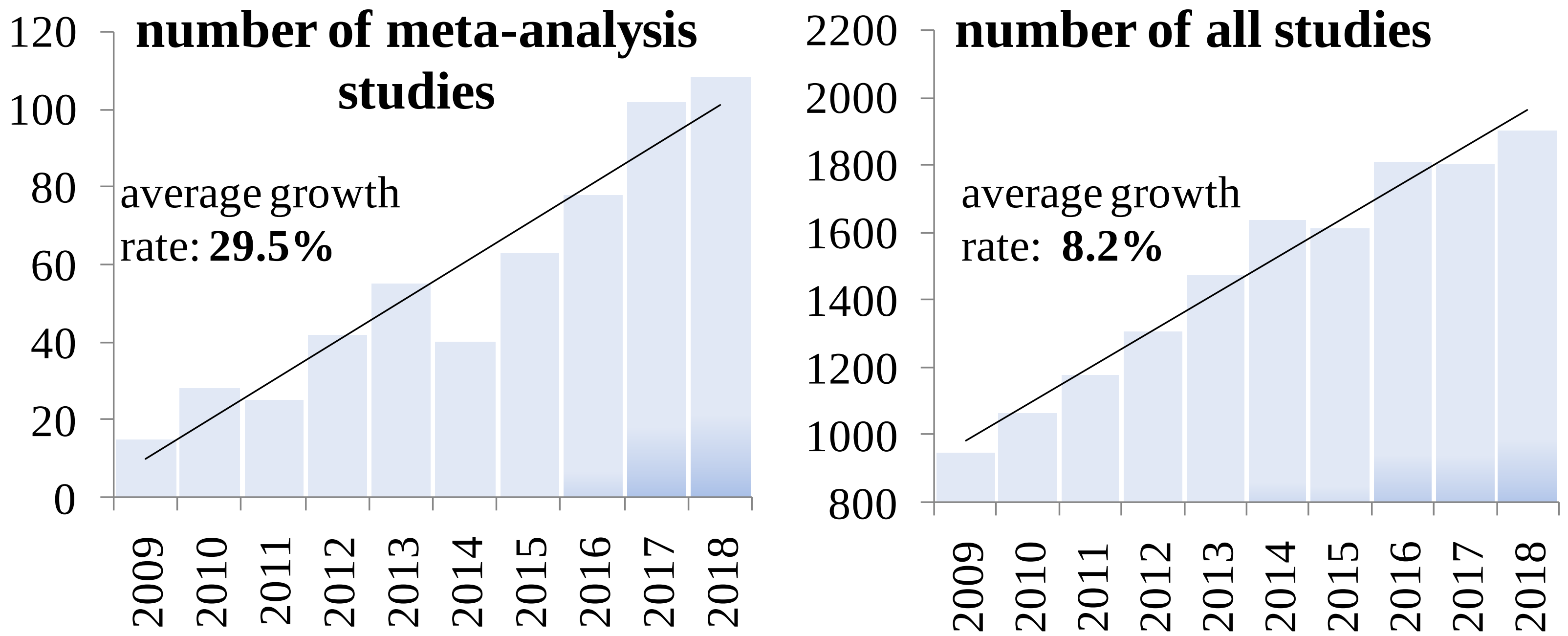


**Fig. S1** The numbers of published papers with meta-analyses related to soil carbon and all studies related to soil carbon from 2009 to 2018.

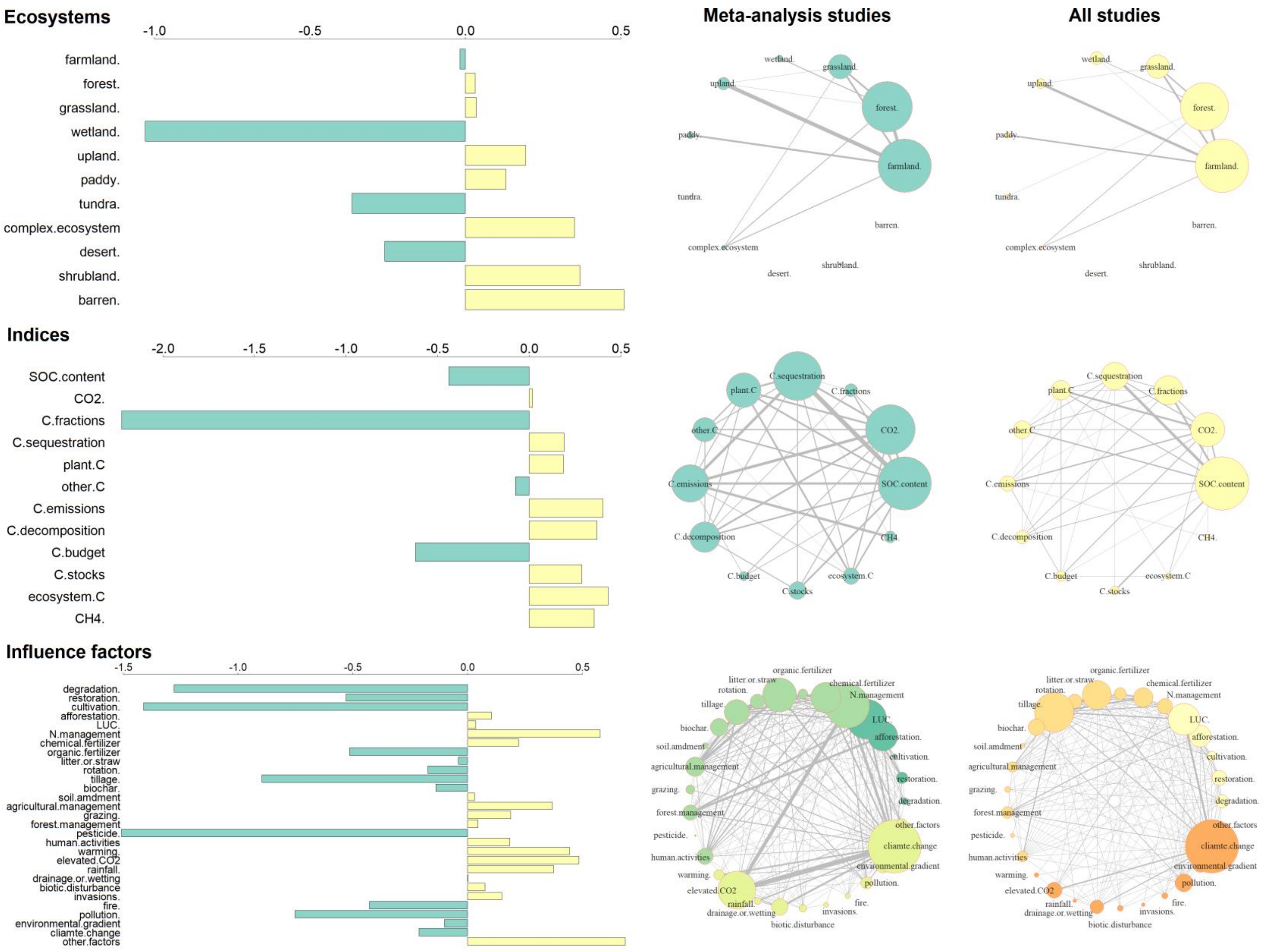
Ecosystems
Meta-analysis studies
All studies
-1.0
-0.5
0.0
0.5
farmland.
forest.
grassland.
wetland.
upland.
paddy.
tundra.
complex.ecosystem
desert.
shrubland.
barren.
Indices
-2.0
-1.5
-1.0
-0.5
0.0
0.5
SOC.content
CO2.
C.fractions
C.sequestration
plant.C
other.C
C.emissions
C.decomposition
C.budget
C.stocks
ecosystem.C
CH4.
Influence factors
-1.5
-1.0
-0.5
0.0
0.5
degradation.
restoration.
cultivation.
afforestation.
LUC.
N.management
chemical.fertilizer
organic.fertilizer
litter.or.straw
rotation.
tillage.
biochar.
soil.amdment
agricultural.management
grazing.
forest.management
pesticide.
human.activities
warming.
elevated.CO2
rainfall.
drainage.or.wetting
biotic.disturbance
invasions.
fire.
pollution.
environmental.gradient
cliamte.change
other.factors

**Fig. S2** Keyword distributions of meta-analyses and all studies related to soil carbon. The bar plot indicates the percentage changes in keyword distributions in the 11 ecosystems, 12 indices and 29 influencing factors between the meta-analyses and all studies related to soil carbon. The network indicates the keyword distributions in the 11 ecosystems, 12 indices and 29 influencing factors and the co-occurrence frequency of two groups; the vertex indicates the relative percentage of each group; the numerical value of the vertex of each group = 50 * keyword count of each group/ the maximum keyword count; and the edges indicate the co-occurrence frequency of two groups. The numerical value of the edge of each group = 50 * co-occurrence frequency of each pair of two groups/ the maximum co-occurrence frequency.

**Table S1** Changes in C losses and C assimilation due to different influencing factors.

| Influencing factor | Changes in C emission | | | | | Changes in C assimilation | | | | |
|---|---|---|---|---|---|---|---|---|---|---|
| | Mean change | Confidence interval of 95% | | Observations | Studies | Mean change | Confidence interval of 95% | | Observations | Studies |
| degradation | -25.8% | -43.6% | -8.1% | 236 | 2 | -16.8% | -25.5% | -8.2% | 133 | 2 |
| forestation | 37.0% | NA | NA | 22 | 1 | NA | NA | NA | NA | NA |
| deforestation | -4.7% | -56.7% | 47.3% | 379 | 3 | NA | NA | NA | NA | NA |
| N addition | 7.8% | -0.9% | 16.5% | 4836 | 13 | 19.9% | 13.8% | 26.0% | 3233 | 21 |
| chemical fertilizer | 113.0% | NA | NA | 10 | 1 | 35.8% | -16.2% | 87.8% | 410 | 2 |
| organic fertilizer | 44.1% | 21.0% | 67.2% | 807 | 3 | 43.0% | NA | NA | 271 | 1 |
| litter input | 67.1% | 37.3% | 96.9% | 846 | 5 | NA | NA | NA | NA | NA |
| unfertilized | -34.3% | -48.0% | -20.6% | 2262 | 2 | NA | NA | NA | NA | NA |
| no tillage | -21.2% | -38.1% | -4.3% | 669 | 5 | 15.4% | NA | NA | 22 | 1 |
| tillage | 21.0% | NA | NA | 174 | 1 | NA | NA | NA | NA | NA |
| soil amendment | -0.3% | -19.4% | 18.7% | 2389 | 11 | 9.6% | -55.7% | 74.9% | 1204 | 4 |
| combined agricultural managements | 18.5% | -60.0% | 97.0% | 67 | 3 | NA | NA | NA | NA | NA |
| reduced grazing | NA | NA | NA | NA | NA | 55.1% | -321.0% | 431.2% | 117 | 1 |
| grazing | 5.0% | -48.0% | 58.1% | 128 | 3 | -29.0% | -49.4% | -8.6% | 476 | 5 |
| forest harvesting | 29.4% | NA | NA | 217 | 1 | -33.6% | -158.7% | 91.6% | 191 | 2 |
| other management | -24.1% | -32.5% | -15.8% | 120 | 2 | NA | NA | NA | NA | NA |
| warming | 16.1% | 4.8% | 27.5% | 1880 | 16 | 11.3% | 5.3% | 17.3% | 1902 | 9 |
| elevated $CO_2$ | 21.1% | 10.7% | 31.5% | 671 | 8 | 25.7% | 19.8% | 31.5% | 4631 | 15 |
| rainfall increased | 26.1% | -22.4% | 74.6% | 371 | 7 | 5.1% | -16.8% | 27.0% | 135 | 4 |
| rainfall reduction | -14.4% | -23.1% | -5.6% | 200 | 6 | -10.3% | -34.4% | 13.7% | 127 | 3 |

| | | | | | | | | | | |
|---|---|---|---|---|---|---|---|---|---|---|
| wetting | 27.9% | -202.8% | 258.5% | 118 | 2 | 26.3% | 13.5% | 39.2% | 179 | 1 |
| drying | -41.7% | -60.3% | -23.0% | 840 | 6 | -24.7% | -31.0% | -18.4% | 519 | 2 |
| biotic disturbance | 6.3% | NA | NA | 15 | 1 | 4.6% | -14.9% | 24.0% | 863 | 2 |
| plant invasion | 116.8% | NA | NA | 58 | 1 | 96.5% | -6.0% | 199.0% | 273 | 3 |
| fire | -13.5% | NA | NA | 139 | 1 | NA | NA | NA | NA | NA |
| pollution | -7.1% | NA | NA | 67 | 1 | -45.7% | -79.7% | -11.8% | 139 | 1 |
| other environmental and climatic change | 1.4% | -12.3% | 15.1% | 512 | 5 | -6.6% | -36.8% | 23.7% | 47 | 2 |

The C losses include $CO_2$ emissions, $CH_4$ emissions and carbon decomposition. C assimilation includes underground biomass, aboveground biomass, plant biomass, net primary production and so on. NA means that no data were reported in the studies or that there were not enough data for the t-tests.

**Table S2** List of the references that the meta-analysis results were collected.

| References related to soil C changes | | References related to soil C emission | References related to soil C assimilation |
|---|---|---|---|
| [1] Abdalla, K. et al. 2016 | [70] Lu, M. et al. 2011 | [1] Abdalla, K. et al. 2016 | [1] Ainsworth, E. A.et al. 2002 |
| [2] Abdalla, M. 2018 | [71] Lu, M. et al. 2013 | [2] Abdalla, M. 2016 | [2] Bejarano-Castillo, M. et al. |
| [3] Achat, D. L. 2015 et al. | [72] Lu, Y. H. et al. 2008 | [3] Aerts, R. 2006 | 2015 |
| [4] Aguilera, E. et al. 2013 | [73] Luo, G. W. et al. 2018 | [4] Amani, M. et al. 2019 | [3] Biederman, L. A.and |
| [5] Alvarez, R. et al. 2017 | [74] Luo, Y. Q. et al. 2006 | [5] Awad, Y. M.et al. 2018 | Harpole, W. S.2013 |
| [6] Angers, D. A. and | [75] Luo, Z. K. et al. 2010 | [6] Banger, K.et al. 2012 | [4] Bouskill, N. J.et al. 2014 |
| Eriksen-Hamel, N. S., 2008 | [76] Ma, D. D. et al. 2018 | [7] Bouskill, N. J.et al. 2014 | [5] Davidson, I. C. et al. 2018 |
| [7] Barcena, T. G.et al. 2014 | [77] Maia, S. M. F. et al. 2013 | [8] Canarini, A. et al. 2017 | [6] Davidson, K. E. et al. 2017 |
| [8] Berthrong, S. T. et al. 2009 | [78] Maillard, E. and Angers, D. A. | [9] Chen, J. et al. 2018 a | [7] de Graaff, M. A. et al. 2006 |
| [9] Biederman, L. A.and Harpole, | 2014 | [10] Chen, X. L. and Chen, H. Y. H., | [8] de Graaff, M. A. et al. 2015 |
| W. S.2013 | [79] Manley, J. et al. 2005 | 2018 | [9] De Schrijver, A. et al. 2011 |
| [10] Bonnesoeur, V. et al. 2019 | [80] Martin, P. A. et al. 2017 | [11] Cong, W. W. et al. 2018 | [10] Deng, L. et al. 2018 |
| [11] Bouskill, N. J.et al. 2014 | [81] McDaniel, M. D. et al. 2014 | [12] de Graaff, M. A. et al. 2014 | [11] Dieleman, W. I. J. et al. |
| [12] Byrnes, R. C. et al. 2018 | [82] Moreno-Mateos, D. et al. 2012 | [13] de Graaff, M. A. et al. 2015 | 2010 |
| [13] Chatterjee, N. et al. 2018 | [83] Nave, L. E. et al. 2009 | [14] Deng, L. et al. 2018 | [12] Dong, Y. L. et al. 2018 |
| [14] Chen, H. et al. 2018 | [84] Nave, L. E. et al. 2010 | [15] Dieleman, W. I. J. et al. 2010 | [13] Fu, G. and Shen, Z. X., 2016 |
| [15] Chen, J. et al. 2018 b | [85] Nave, L. E. et al. 2011 | [16] Feng, J. F. et al. 2018 | [14] Fu, Z. et al. 2015 |
| [16] Chen, Y. S. et al. 2018 | [86] Nave, L. E. et al. 2013 | [17] Feng, J. G. et al. 2017 | [15] Gao, W. L. and Yan, D. H., |
| [17] Cheng, L. et al. 2017 | [87] Nayak, D. et al. 2015 | [18] Fu, Z. et al. 2015 | 2019 |
| [18] Congreves, K. A. et al., 2014 | [88] Nyawira, S. S. et al. 2016 | [19] Gao, W. L. and Yan, D. H., 2019 | [16] Garcia-Palacios, P. et al. |
| [19] Dai, Z. M. et al. 2018 | [89] Obade, V. D. and Lal, R. 2014 | [20] Garcia-Palacios, P. et al. 2018 | 2015 |
| [20] Davidson, I. C. et al. 2018 | [90] Ogle, S. M. et al. 2005 | [21] He, Y. H. et al. 2017 | [17] Gravuer, K. et al. 2019 |

| | | | |
|---|---|---|---|
| [21] Davidson, K. E. et al. 2017 | [91] Olsson, M. T. et al. 2009 | [22] Hergoualc'h, K. and Verchot, L. V. | [18] Janssens, I. A. et al. 2010 |
| [22] de Graaff, M. A. et al. 2006 | [92] Peng, X. et al. 2017 | 2012 | [19] Ji, C. et al. 2018 |
| [23] de Graaff, M. A. et al. 2014 | [93] Powers, J. S. et al. 2011 | [23] Hergoualc'h, K. and Verchot, L. V. | [20] Kivlin, S. N. et al. 2013 |
| [24] De Stefano, A. and Jacobson, | [94] Qin, Z. C. et al. 2016 | 2014 | [21] LeBauer, D. S. and |
| M. G. 2018 | [95] Ren, C. J. et al. 2017 | [24] Huang, Y. W. et al. 2018 | Treseder, K. K. 2008 |
| [25] Deng, L. et al. 2018 | [96] Ren, C. J. et al. 2018 | [25] Janssens, I. A. et al. 2010 | [22] Lee, M. et al. 2010 |
| [26] Dieleman, W. I. J. et al. 2010 | [97] Ribeiro, A. A. et al. 2015 | [26] Jeffery, S. et al. 2016 | [23] Li, W. B. et al. 2015 |
| [27] Dlamini, P. et al. 2016 | [98] Shi, L. L. et al. 2018 | [27] Ji, C. et al. 2018 | [24] Liao, C. Z. et al. 2008 |
| [28] Don, A. et al. 2011 | [99] Shi, S. W. et al. 2013 | [28] Jiang, Y. et al. 2019 | [25] Liao, C. Z. et al. 2010 |
| [29] Du, Z. L. et al. 2017 | [100] Shi, S. W. et al. 2016 | [29] Li, W. B. et al. 2016 | [26] Lin, D. L. et al. 2010 |
| [30] Dybala, K. E. et al. 2019 | [101] Sileshi, G. W. 2016 | [30] Liao, C. Z. et al. 2008 | [27] Liu, L. L. and Greaver, T. |
| [31] Eze, S. et al. 2018 | [102] Sileshi, G. W. et al. 2010 | [31] Liao, C. Z. et al. 2010 | L., 2009 |
| [32] Felix, G. F. et al. 2018 | [103] Sillen, W. M. A. and | [32] Liu, C. et al. 2014 | [28] Liu, L. L. and Greaver, T. |
| [33] Fu, Z. et al. 2015 | Dieleman, W. I. J. 2012 | [33] Liu, L. L. and Greaver, T. L., 2009 | L., 2010 |
| [34] Fujisaki, K. et al. 2017 | [104] Song, X. Z. et al. 2014 | [34] Liu, L. L. et al. 2016 | [29] Liu, S. W. et al.2018 |
| [35] Gao, W. L. and Yan, D. H., | [105] Swanepoel, C. M. et al. | [35] Liu, S. W. et al.2015 | [30] Lu, M. et al. 2011 |
| 2019 | 2016 | [36] Liu, S. W. et al.2018 | [31] Lu, M. et al. 2013 |
| [36] Garcia-Palacios, P. et al. 2018 | [106] Tang, S. M. et al. 2018 | [37] Lu, M. et al. 2013 | [32] Luo, Y. Q. et al. 2006 |
| [37] Geisseler, D. and Scow, K. M. | [107] Tang, S. M. et al. 2019a | [38] Nayak, D. et al. 2015 | [33] Martin, P. A. et al. 2017 |
| 2014 | [108] Tang, S. M. et al. 2019b | [39] Ren, C. J. et al. 2017 | [34] Nie, M. et al. 2013 |
| [38] Geisseler, D. et al. 2017 | [109] Tian, D. S. et al. 2018 | [40] Ren, C. J. et al. 2018 | [35] Peng, X. et al. 2017 |
| [39] Gong, L. er al. 2017 | [110] Van Groenigen, J. W. et al. | [41] Romero-Olivares, A. L. et al. 2017 | [36] Peng, Y. F. et al. 2017 |
| [40] Grace, P. R. et al. 2012 | 2019 | [42] Sagrilo, E. et al. 2015 | [37] Peng, Y. F. et al. 2019 |
| [41] Gravuer, K. et al. 2019 | [111] van Groenigen, K. J. et al. | [43] Sainju, U. M. et al. 2016 | [38] Schulte-Uebbing, L. and de |
| [42] Guo, L. B. and Gifford, R. M. | 2017 | [44] Schadel, C. et al. 2016 | Vries, W. 2018 |
| 2002 | [112] Vicente-Vicente, J. L. et | [45] Skinner, C. et al. 2014 | [39] Sillen, W. M. A. and |

| | | | |
|---|---|---|---|
| [43] Han, P. F. et al. 2016 | al. 2016 | [46] Song, X. Z. et al. 2013 | Dieleman, W. I. J. 2012 |
| [44] Harris, Z. M. et al. 2015 | [113] Virto, I. et al. 2012 | [47] Song, Y. et al. 2017 | [40] Song, Y. et al. 2017 |
| [45] Huang, S. et al. 2012 | [114] Wan, X. H. et al. 2018 | [48] Sun, J. F. et al. 2011 | [41] Tang, S. M. et al. 2018 |
| [46] Hume, A. M. et al. 2018 | [115] Wang, Q. K. et al. 2012 | [49] Tang, S. M. et al. 2019b | [42] Tang, S. M. et al. 2019b |
| [47] James, J. and Harrison, R., | [116] Wang, X. Y. et al. 2016 | [50] van Groenigen, K. J. et al. 2011 | [43] Thomas, S. C. and Gale, N. |
| 2016 | [117] Wei, H. et al. 2017 | [51] van Groenigen, K. J. et al. 2013 | 2015 |
| [48] Janssens, I. A. et al. 2010 | [118] Xia, L. L. et al. 2017 | [52] van Groenigen, K. J. et al. 2014 | [44] Tian, D. H. 2016 et al. |
| [49] Jastrow, J. D. et al. 2005 | [119] Xia, L. L. et al. 2018 | [53] Wang, J. et al. 2015 | [45] van der Kooi, C. J. et al. |
| [50] Jian, S. Y. et al. 2016 | [120] Xiong, D. P. et al. 2016 | [54] Wang, J. Y. et al. 2016 | 2016 |
| [51] Johnson, D. W. and Curtis, P. | [121] Xu, S. et al. 2013 | [55] Wang, Q. K. et al. 2012 | [46] van Groenigen, J. W. et al. |
| S., 2001 | [122] Yan, G. Y. et al. 2018 | [56] Wang, X. et al. 2014 | 2014 |
| [52] Kampf, I. et al. 2016 | [123] Yu, L. F. et al. 2017 | [57] Wang, X. Y. et al. 2016 | [47] van Groenigen, K. J. et al. |
| [53] Kaschuk, G. et al. 2011 | [124] Yuan, Z. Y. et al. 2017 | [58] Wu, Z. T. et al. 2011 | 2013 |
| [54] King, A. E. and Blesh, J., 2018 | [125] Yue, K. et al. 2016 | [59] Xia, L. L. et al. 2017 | [48] Wang, X. Z. 2007 |
| [55] Kopittke, P. M. et al. 2017 | [126] Zhang, B. C. et al. 2015 | [60] Xu, S. et al. 2013 | [49] Wu, Z. T. et al. 2011 |
| [56] Ladha, J. K. et all. 2011 | [127] Zhang, X. Z. et al. 2015 | [61] Yan, G. Y. et al. 2018 | [50] Xiang, Y. Z. et al. 2017 |
| [57] Laganiere, J. et al. 2010 | [128] Zhang, X. Z. et al. 2018 | [62] Yue, K. et al. 2015 | [51] Xiong, D. P. et al. 2016 |
| [58] Li, H. et al. 2016 | [129] Zhao, H. et al. 2015 | [63] Yue, K. et al. 2016 | [52] Yan, G. Y. et al. 2018 |
| [59] Li, W. B. et al. 2016 | [130] Zhao, H. et al. 2017 | [64] Zhang, B. C. et al. 2015 | [53] Yan, L. et al. 2013 |
| [60] Li, Y. E. et al. 2018 | [131] Zhou, D. et al. 2013 | [65] Zhang, T. A. et al. 2018 | [54] Yendrek, C. R. et al. 2013 |
| [61] Liao, C. Z. et al. 2008 | [132] Zhou, G. Y. et al. 2017a | [66] Zhang, W. D. et al. 2013 | [55] Yue, K. et al. 2016 |
| [62] Liao, C. Z. et al. 2010 | [133] Zhou, T. et al. 2016 | [67] Zhang, X. Z. et al. 2018 | [56] Zhang, B. C. et al. 2015 |
| [63] Liao, C. Z. et al. 2012 | [134] Zhou, X. H. et al. 2016 | [68] Zhao, X. et al. 2016 | [57] Zhang, X. Z. et al. 2018 |
| [64] Liu, C. et al. 2014 | [135] Zhou, Z. H. et al. 2018b | [69] Zhao, X. et al. 2019 | [58] Zhao, X. et al. 2016 |
| [65] Liu, L. L. and Greaver, T. L., | [136] Zhou, Z. H. et al. 2018a | [70] Zhong, Y. Q. W. et al. 2016 | [59] Zheng, F. Y. and Peng, S. L. |
| 2009 | [137] Zhu, L. Q. et al. 2015 | [71] Zhou, G. Y. et al. 2017a | 2001 |

| | | | |
|---|---|---|---|
| [66] Liu, L. L. and Greaver, T. L., 2010 | [138] Zinn, Y. L. et al. 2005 | [72] Zhou, G. Y. et al. 2017b | [60] Zhou, D. et al. 2013 |
| | | [73] Zhou, L. Y. et al. 2014 | [61] Zhou, G. Y. et al. 2017a |
| [67] Liu, S. W. et al.2015 | | [74] Zhou, L. Y. et al. 2016 | [62] Zhou, T. et al. 2016 |
| [68] Liu, S. W. et al.2018 | | [75] Zhou, Z. H. et al. 2018b | [63] Zhou, X. H. et al. 2016 |
| [69] Liu, X. et al.2018 | | [76] Zhou, Z. H. et al. 2018a | [64] Zhou, Z. H. et al. 2018a |

**References**


[1] Abdalla, K., Chivenge, P., Ciais, P., & Chaplot, V. (2016). No-tillage lessens soil CO2 emissions the most under arid and sandy soil conditions: results from a meta-analysis. *Biogeosciences, 13*(12), 3619-3633. doi:10.5194/bg-13-3619-2016

[2] Abdalla, M., Hastings, A., Chadwick, D. R., Jones, D. L., Evans, C. D., Jones, M. B., . . . Smith, P. (2018). Critical review of the impacts of grazing intensity on soil organic carbon storage and other soil quality indicators in extensively managed grasslands. *Agriculture Ecosystems & Environment, 253,* 62-81. doi:10.1016/j.agee.2017.10.023

[3] Abdalla, M., Hastings, A., Truu, J., Espenberg, M., Mander, U., & Smith, P. (2016). Emissions of methane from northern peatlands: a review of management impacts and implications for future management options. *Ecology and Evolution, 6*(19), 7080-7102. doi:10.1002/ece3.2469

[4] Achat, D. L., Fortin, M., Landmann, G., Ringeval, B., & Augusto, L. (2015). Forest soil carbon is threatened by intensive biomass harvesting. *Scientific Reports, 5,* 10. doi:10.1038/srep15991

[5] Aerts, R. (2006). The freezer defrosting: global warming and litter decomposition rates in cold biomes. *Journal of Ecology, 94*(4), 713-724. doi:10.1111/j.1365-2745.2006.01142.x

[6] Aguilera, E., Lassaletta, L., Gattinger, A., & Gimeno, B. S. (2013). Managing soil carbon for climate change mitigation and adaptation in Mediterranean cropping systems: A meta-analysis. *Agriculture Ecosystems & Environment, 168,* 25-36. doi:10.1016/j.agee.2013.02.003

[7] Ainsworth, E. A., Davey, P. A., Bernacchi, C. J., Dermody, O. C., Heaton, E. A., Moore, D. J., . . . Long, S. P. (2002). A meta-analysis of elevated CO2 effects on soybean (Glycine max) physiology, growth and yield. *Global Change Biology, 8*(8), 695-709. doi:10.1046/j.1365-2486.2002.00498.x

[8] Alvarez, R., Steinbach, H. S., & De Paepe, J. L. (2017). Cover crop effects on soils and subsequent crops in the pampas: A meta-analysis. *Soil & Tillage Research, 170,* 53-65. doi:10.1016/j.still.2017.03.005

[9] Amani, M., Graca, M. A. S., & Ferreira, V. (2019). Effects of elevated atmospheric CO2 concentration and temperature on litter decomposition in streams: A meta-analysis. *International Review of Hydrobiology, 104*(1-2), 14-25. doi:10.1002/iroh.201801965

[10] Angers, D. A., & Eriksen-Hamel, N. S. (2008). Full-inversion tillage and organic carbon distribution in soil profiles: A meta-analysis. *Soil Science Society of America Journal, 72*(5), 1370-1374. doi:10.2136/sssaj2007.0342

[11] Awad, Y. M., Wang, J. Y., Igalavithana, A. D., Tsang, D. C. W., Kim, K. H., Lee, S. S., & Ok, Y. S. (2018). Biochar Effects on Rice Paddy: Meta-analysis. In D. L. Sparks (Ed.), *Advances in Agronomy, Vol 148* (Vol. 148, pp. 1-32). San Diego: Elsevier Academic Press Inc.

[12] Banger, K., Tian, H. Q., & Lu, C. Q. (2012). Do nitrogen fertilizers stimulate or inhibit methane emissions from rice fields? *Global Change Biology, 18*(10), 3259-3267. doi:10.1111/j.1365-2486.2012.02762.x

[13] Barcena, T. G., Kiaer, L. P., Vesterdal, L., Stefansdottir, H. M., Gundersen, P., & Sigurdsson, B. D. (2014). Soil carbon stock change following afforestation in Northern Europe: a meta-analysis. *Global Change Biology, 20*(8), 2393-2405. doi:10.1111/gcb.12576

[14] Bejarano-Castillo, M., Campo, J., & Roa-Fuentes, L. L. (2015). Effects of Increased Nitrogen Availability on C and N Cycles in Tropical Forests: A Meta-Analysis. *Plos One, 10*(12), 12. doi:10.1371/journal.pone.0144253

[15] Berthrong, S. T., Jobbagy, E. G., & Jackson, R. B. (2009). A global meta-analysis of soil exchangeable cations, pH, carbon, and nitrogen with afforestation. *Ecological Applications, 19*(8), 2228-2241. doi:10.1890/08-1730.1

[16] Biederman, L. A., & Harpole, W. S. (2013). Biochar and its effects on plant productivity and nutrient cycling: a meta-analysis. *Global Change Biology Bioenergy, 5*(2), 202-214. doi:10.1111/gcbb.12037

[17] Bonnesoeur, V., Locatelli, B., Guariguata, M. R., Ochoa-Tocachi, B. F., Vanacker, V., Mao, Z., . . . Mathez-Stiefel, S. L. (2019). Impacts of forests and forestation on hydrological services in the Andes: A systematic review. *Forest Ecology and Management, 433*, 569-584. doi:10.1016/foreco.2018.11.033

[18] Bouskill, N. J., Riley, W. J., & Tang, J. Y. (2014). Meta-analysis of high-latitude nitrogen-addition and warming studies implies ecological mechanisms overlooked by land models. *Biogeosciences, 11*(23), 6969-6983. doi:10.5194/bg-11-6969-2014

[19] Byrnes, R. C., Eastburn, D. J., Tate, K. W., & Roche, L. M. (2018). A Global Meta-Analysis of Grazing Impacts on Soil Health Indicators. *Journal of Environmental Quality, 47*(4), 758-765. doi:10.2134/jeq2017.08.0313

[20] Canarini, A., Kiaer, L. P., & Dijkstra, F. A. (2017). Soil carbon loss regulated by drought intensity and available substrate: A meta-analysis. *Soil Biology & Biochemistry, 112*, 90-99. doi:10.1016/j.soilbio.2017.04.020

[21] Chatterjee, N., Nair, P. K. R., Chakraborty, S., & Nair, V. D. (2018). Changes in soil carbon stocks across the Forest-Agroforest-Agriculture/Pasture continuum in various agroecological regions: A meta-analysis. *Agriculture Ecosystems & Environment, 266*, 55-67. doi:10.1016/j.agee.2018.07.014

[22] Chen, H., Li, D., Feng, W., Niu, S., Plante, A. F., Luo, Y., & Wang, K. (2018). Different responses of soil organic carbon fractions to additions of nitrogen. *European Journal of Soil Science, 69*(6), 1098-1104. doi:10.1111/ejss.12716

[23] Chen, J., Luo, Y. Q., Garcia-Palacios, P., Cao, J. J., Dacal, M., Zhou, X. H., . . . van Groenigen, K. J. (2018). Differential responses of carbon-degrading enzyme activities to warming: Implications for soil respiration. *Global Change Biology, 24*(10), 4816-4826. doi:10.1111/gcb.14394

[24] Chen, J., Luo, Y. Q., van Groenigen, K. J., Hungate, B. A., Cao, J. J., Zhou, X. H., & Wang, R. W. (2018). A keystone microbial enzyme for nitrogen control of soil carbon storage. *Science Advances, 4*(8), 6. doi:10.1126/sciadv.aaq1689

[25] Chen, X. L., & Chen, H. Y. H. (2018). Global effects of plant litter alterations on soil CO2 to the atmosphere. *Global Change Biology, 24*(8), 3462-3471. doi:10.1111/gcb.14147

[26] Chen, Y. S., Camps-Arbestain, M., Shen, Q. H., Singh, B., & Cayuela, M. L. (2018). The long-term role of organic amendments in building soil nutrient fertility: a meta-analysis and review. *Nutrient Cycling in Agroecosystems, 111*(2-3), 103-125. doi:10.1007/s10705-017-9903-5
[27] Cheng, L., Zhang, N. F., Yuan, M. T., Xiao, J., Qin, Y. J., Deng, Y., . . . Zhou, J. Z. (2017). Warming enhances old organic carbon decomposition through altering functional microbial communities. *Isme Journal, 11*(8), 1825-1835. doi:10.1038/ismej.2017.48
[28] Cong, W. W., Meng, J., & Ying, S. C. (2018). Impact of soil properties on the soil methane flux response to biochar addition: a meta-analysis. *Environmental Science-Processes & Impacts, 20*(9), 1202-1209. doi:10.1039/c8em00278a
[29] Congreves, K. A., Smith, J. M., Nemeth, D. D., Hooker, D. C., & Van Eerd, L. L. (2014). Soil organic carbon and land use: Processes and potential in Ontario's long-term agro-ecosystem research sites. *Canadian Journal of Soil Science, 94*(3), 317-336. doi:10.4141/cjss2013-094
[30] Dai, Z. M., Su, W. Q., Chen, H. H., Barberan, A., Zhao, H. C., Yu, M. J., . . . Xu, J. M. (2018). Long-term nitrogen fertilization decreases bacterial diversity and favors the growth of Actinobacteria and Proteobacteria in agro-ecosystems across the globe. *Global Change Biology, 24*(8), 3452-3461. doi:10.1111/gcb.14163
[31] Davidson, I. C., Cott, G. M., Devaney, J. L., & Simkanin, C. (2018). Differential effects of biological invasions on coastal blue carbon: A global review and meta-analysis. *Global Change Biology, 24*(11), 5218-5230. doi:10.1111/gcb.14426
[32] Davidson, K. E., Fowler, M. S., Skov, M. W., Doerr, S. H., Beaumont, N., & Griffin, J. N. (2017). Livestock grazing alters multiple ecosystem properties and services in salt marshes: a meta-analysis. *Journal of Applied Ecology, 54*(5), 1395-1405. doi:10.1111/1365-2664.12892
[33] de Graaff, M. A., Adkins, J., Kardol, P., & Throop, H. L. (2015). A meta-analysis of soil biodiversity impacts on the carbon cycle. *Soil, 1*(1), 257-271. doi:10.5194/soil-1-257-2015
[34] de Graaff, M. A., Throop, H. L., Verburg, P. S. J., Arnone, J. A., & Campos, X. (2014). A Synthesis of Climate and Vegetation Cover Effects on Biogeochemical Cycling in Shrub-Dominated Drylands. *Ecosystems, 17*(5), 931-945. doi:10.1007/s10021-014-9764-6
[35] de Graaff, M. A., van Groenigen, K. J., Six, J., Hungate, B., & van Kessel, C. (2006). Interactions between plant growth and soil nutrient cycling under elevated CO2: a meta-analysis. *Global Change Biology, 12*(11), 2077-2091. doi:10.1111/j.1365-2486.2006.01240.x
[36] De Schrijver, A., De Frenne, P., Ampoorter, E., Van Nevel, L., Demey, A., Wuyts, K., & Verheyen, K. (2011). Cumulative nitrogen input drives species loss in terrestrial ecosystems. *Global Ecology and Biogeography, 20*(6), 803-816. doi:10.1111/j.1466-8238.2011.00652.x
[37] De Stefano, A., & Jacobson, M. G. (2018). Soil carbon sequestration in agroforestry systems: a meta-analysis. *Agroforestry Systems, 92*(2), 285-299. doi:10.1007/s10457-017-0147-9
[38] Deng, L., Peng, C. H., Zhu, G. Y., Chen, L., Liu, Y. L., & Shangguan, Z. P. (2018). Positive responses of belowground C dynamics to nitrogen

enrichment in China. *Science of the Total Environment, 616*, 1035-1044. doi:10.1016/j.scitotenv.2017.10.215
[39] Dieleman, W. I. J., Luyssaert, S., Rey, A., De Angelis, P., Barton, C. V. M., Broadmeadow, M. S. J., . . . Janssens, I. A. (2010). Soil N modulates soil C cycling in CO2-fumigated tree stands: a meta-analysis. *Plant Cell and Environment, 33*(12), 2001-2011. doi:10.1111/j.1365-3040.2010.02201.x
[40] Dlamini, P., Chivenge, P., & Chaplot, V. (2016). Overgrazing decreases soil organic carbon stocks the most under dry climates and low soil pH: A meta-analysis shows. *Agriculture Ecosystems & Environment, 221*, 258-269. doi:10.1016/j.agee.2016.01.026
[41] Don, A., Schumacher, J., & Freibauer, A. (2011). Impact of tropical land-use change on soil organic carbon stocks - a meta-analysis. *Global Change Biology, 17*(4), 1658-1670. doi:10.1111/j.1365-2486.2010.02336.x
[42] Dong, Y. L., Wang, Z. Y., Sun, H., Yang, W. C., & Xu, H. (2018). The Response Patterns of Arbuscular Mycorrhizal and Ectomycorrhizal Symbionts Under Elevated CO2: A Meta-Analysis. *Frontiers in Microbiology, 9*, 14. doi:10.3389/fmicb.2018.01248
[43] Du, Z. L., Angers, D. A., Ren, T. S., Zhang, Q. Z., & Li, G. C. (2017). The effect of no-till on organic C storage in Chinese soils should not be overemphasized: A meta-analysis. *Agriculture Ecosystems & Environment, 236*, 1-11. doi:10.1016/j.agee.2016.11.007
[44] Dybala, K. E., Matzek, V., Gardali, T., & Seavy, N. E. (2019). Carbon sequestration in riparian forests: A global synthesis and meta-analysis. *Global Change Biology, 25*(1), 57-67. doi:10.1111/gcb.14475
[45] Eze, S., Palmer, S. M., & Chapman, P. J. (2018). Soil organic carbon stock in grasslands: Effects of inorganic fertilizers, liming and grazing in different climate settings. *Journal of Environmental Management, 223*, 74-84. doi:10.1016/j.jenvman.2018.06.013
[46] Felix, G. F., Scholberg, J. M. S., Clermont-Dauphin, C., Cournac, L., & Tittonell, P. (2018). Enhancing agroecosystem productivity with woody perennials in semi-arid West Africa. A meta-analysis. *Agronomy for Sustainable Development, 38*(6), 21. doi:10.1007/s13593-018-0533-3
[47] Feng, J. F., Li, F. B., Zhou, X. Y., Xu, C. C., Ji, L., Chen, Z. D., & Fang, F. P. (2018). Impact of agronomy practices on the effects of reduced tillage systems on CH4 and N2O emissions from agricultural fields: A global meta-analysis. *Plos One, 13*(5), 17. doi:10.1371/journal.pone.0196703
[48] Feng, J. G., Wang, J. S., Ding, L. B., Yao, P. P., Qiao, M. P., & Yao, S. C. (2017). Meta-analyses of the effects of major global change drivers on soil respiration across China. *Atmospheric Environment, 150*, 181-186. doi:10.1016/j.atmosenv.2016.11.060
[49] Fu, G., & Shen, Z. X. (2016). Response of Alpine Plants to Nitrogen Addition on the Tibetan Plateau: A Meta-analysis. *Journal of Plant Growth Regulation, 35*(4), 974-979. doi:10.1007/s00344-016-9595-0
[50] Fu, Z., Niu, S. L., & Dukes, J. S. (2015). What have we learned from global change manipulative experiments in China? A meta-analysis. *Scientific Reports, 5*, 11. doi:10.1038/srep12344
[51] Fujisaki, K., Perrin, A. S., Desjardins, T., Bernoux, M., Balbino, L. C., & Brossard, M. (2015). From forest to cropland and pasture systems: a critical

review of soil organic carbon stocks changes in Amazonia. *Global Change Biology, 21*(7), 2773-2786. doi:10.1111/gcb.12906

[52] Gao, W. L., & Yan, D. H. (2019). Warming suppresses microbial biomass but enhances N recycling. *Soil Biology & Biochemistry, 131*, 111-118. doi:10.1016/j.soilbio.2019.01.002

[53] Garcia-Palacios, P., Gattinger, A., Bracht-Jorgensen, H., Brussaard, L., Carvalho, F., Castro, H., . . . Milla, R. (2018). Crop traits drive soil carbon sequestration under organic farming. *Journal of Applied Ecology, 55*(5), 2496-2505. doi:10.1111/1365-2664.13113

[54] Garcia-Palacios, P., Vandegehuchte, M. L., Shaw, E. A., Dam, M., Post, K. H., Ramirez, K. S., . . . Wall, D. H. (2015). Are there links between responses of soil microbes and ecosystem functioning to elevated CO2, N deposition and warming? A global perspective. *Global Change Biology, 21*(4), 1590-1600. doi:10.1111/gcb.12788

[55] Geisseler, D., Linguist, B. A., & Lazicki, P. A. (2017). Effect of fertilization on soil microorganisms in paddy rice systems - A meta-analysis. *Soil Biology & Biochemistry, 115*, 452-460. doi:10.1016/j.soilbio.2017.09.018

[56] Geisseler, D., & Scow, K. M. (2014). Long-term effects of mineral fertilizers on soil microorganisms - A review. *Soil Biology & Biochemistry, 75*, 54-63. doi:10.1016/j.soilbio.2014.03.023

[57] Gong, L., Liu, G. H., Wang, M., Ye, X., Wang, H., & Li, Z. S. (2017). Effects of vegetation restoration on soil organic carbon in China: A meta-analysis. *Chinese Geographical Science, 27*(2), 188-200. doi:10.1007/s11769-017-0858-x

[58] Grace, P. R., Antle, J., Aggarwal, P. K., Ogle, S., Paustian, K., & Basso, B. (2012). Soil carbon sequestration and associated economic costs for farming systems of the Indo-Gangetic Plain: A meta-analysis. *Agriculture Ecosystems & Environment, 146*(1), 137-146. doi:10.1016/j.agee.2011.10.019

[59] Gravuer, K., Gennet, S., & Throop, H. L. (2019). Organic amendment additions to rangelands: A meta-analysis of multiple ecosystem outcomes. *Global Change Biology, 25*(3), 1152-1170. doi:10.1111/gcb.14535

[60] Guo, L. B., & Gifford, R. M. (2002). Soil carbon stocks and land use change: a meta analysis. *Global Change Biology, 8*(4), 345-360. doi:10.1046/j.1354-1013.2002.00486.x

[61] Han, P. F., Zhang, W., Wang, G. C., Sun, W. J., & Huang, Y. (2016). Changes in soil organic carbon in croplands subjected to fertilizer management: a global meta-analysis. *Scientific Reports, 6*, 12. doi:10.1038/srep27199

[62] Harris, Z. M., Spake, R., & Taylor, G. (2015). Land use change to bioenergy: A meta-analysis of soil carbon and GHG emissions. *Biomass & Bioenergy, 82*, 27-39. doi:10.1016/j.biombioe.2015.05.008

[63] He, Y. H., Zhou, X. H., Jiang, L. L., Li, M., Du, Z. G., Zhou, G. Y., . . . Xu, C. Y. (2017). Effects of biochar application on soil greenhouse gas fluxes: a meta-analysis. *Global Change Biology Bioenergy, 9*(4), 743-755. doi:10.1111/gcbb.12376

[64] Hergoualc'h, K., & Verchot, L. V. (2014). Greenhouse gas emission factors for land use and land-use change in Southeast Asian peatlands. *Mitigation and Adaptation Strategies for Global Change, 19*(6), 789-807. doi:10.1007/s11027-013-9511-x
[65] Hergoualc'h, K. A., & Verchot, L. V. (2012). Changes in soil CH4 fluxes from the conversion of tropical peat swamp forests: a meta-analysis. *Journal of Integrative Environmental Sciences, 9*(2), 93-101. doi:10.1080/1943815x.2012.679282
[66] Huang, S., Sun, Y. N., & Zhang, W. J. (2012). Changes in soil organic carbon stocks as affected by cropping systems and cropping duration in China's paddy fields: a meta-analysis. *Climatic Change, 112*(3-4), 847-858. doi:10.1007/s10584-011-0255-x
[67] Huang, Y. W., Ren, W., Wang, L. X., Hui, D. F., Grove, J. H., Yang, X. J., . . . Goff, B. (2018). Greenhouse gas emissions and crop yield in no-tillage systems: A meta- analysis. *Agriculture Ecosystems & Environment, 268*, 144-153. doi:10.1016/j.agee.2018.09.002
[68] Hume, A. M., Chen, H. Y. H., & Taylor, A. R. (2018). Intensive forest harvesting increases susceptibility of northern forest soils to carbon, nitrogen and phosphorus loss. *Journal of Applied Ecology, 55*(1), 246-255. doi:10.1111/1365-2664.12942
[69] James, J., & Harrison, R. (2016). The Effect of Harvest on Forest Soil Carbon: A Meta-Analysis. *Forests, 7*(12), 22. doi:10.3390/f7120308
[70] Janssens, I. A., Dieleman, W., Luyssaert, S., Subke, J. A., Reichstein, M., Ceulemans, R., . . . Law, B. E. (2010). Reduction of forest soil respiration in response to nitrogen deposition. *Nature Geoscience, 3*(5), 315-322. doi:10.1038/ngeo844
[71] Jastrow, J. D., Miller, R. M., Matamala, R., Norby, R. J., Boutton, T. W., Rice, C. W., & Owensby, C. E. (2005). Elevated atmospheric carbon dioxide increases soil carbon. *Global Change Biology, 11*(12), 2057-2064. doi:10.1111/j.1365-2486.2005.01077.x
[72] Jeffery, S., Verheijen, F. G. A., Kammann, C., & Abalos, D. (2016). Biochar effects on methane emissions from soils: A meta-analysis. *Soil Biology & Biochemistry, 101*, 251-258. doi:10.1016/j.soilbio.2016.07.021
[73] Ji, C., Jin, Y. G., Li, C., Chen, J., Kong, D. L., Yu, K., . . . Zou, J. W. (2018). Variation in Soil Methane Release or Uptake Responses to Biochar Amendment: A Separate Meta-analysis. *Ecosystems, 21*(8), 1692-1705. doi:10.1007/s10021-018-0248-y
[74] Jian, S. Y., Li, J. W., Chen, J., Wang, G. S., Mayes, M. A., Dzantor, K. E., . . . Luo, Y. Q. (2016). Soil extracellular enzyme activities, soil carbon and nitrogen storage under nitrogen fertilization: A meta-analysis. *Soil Biology & Biochemistry, 101*, 32-43. doi:10.1016/j.soilbio.2016.07.003
[75] Jiang, Y., Carrijo, D., Huang, S., Chen, J., Balaine, N., Zhang, W. J., . . . Linquist, B. (2019). Water management to mitigate the global warming potential of rice systems: A global meta-analysis. *Field Crops Research, 234*, 47-54. doi:10.1016/j.fcr.2019.02.010
[76] Johnson, D. W., & Curtis, P. S. (2001). Effects of forest management on soil C and N storage: meta analysis. *Forest Ecology and Management, 140*(2-3), 227-238. doi:10.1016/s0378-1127(00)00282-6
[77] Kampf, I., Holzel, N., Storrle, M., Broll, G., & Kiehl, K. (2016). Potential of temperate agricultural soils for carbon sequestration: A meta-analysis of

land-use effects. *Science of the Total Environment*, *566*, 428-435. doi:10.1016/j.scitotenv.2016.05.067

[78] Kaschuk, G., Alberton, O., & Hungria, M. (2011). Quantifying effects of different agricultural land uses on soil microbial biomass and activity in Brazilian biomes: inferences to improve soil quality. *Plant and Soil*, *338*(1-2), 467-481. doi:10.1007/s11104-010-0559-z

[79] King, A. E., & Blesh, J. (2018). Crop rotations for increased soil carbon: perenniality as a guiding principle. *Ecological Applications, 28*(1), 249-261. doi:10.1002/eap.1648/full

[80] Kivlin, S. N., Emery, S. M., & Rudgers, J. A. (2013). FUNGAL SYMBIONTS ALTER PLANT RESPONSES TO GLOBAL CHANGE. *American Journal of Botany, 100*(7), 1445-1457. doi:10.3732/ajb.1200558

[81] Kopittke, P. M., Dalal, R. C., Finn, D., & Menzies, N. W. (2017). Global changes in soil stocks of carbon, nitrogen, phosphorus, and sulphur as influenced by long-term agricultural production. *Global Change Biology, 23*(6), 2509-2519. doi:10.1111/gcb.13513

[82] Ladha, J. K., Reddy, C. K., Padre, A. T., & van Kessel, C. (2011). Role of Nitrogen Fertilization in Sustaining Organic Matter in Cultivated Soils. *Journal of Environmental Quality, 40*(6), 1756-1766. doi:10.2134/jeq2011.0064

[83] Laganiere, J., Angers, D. A., & Pare, D. (2010). Carbon accumulation in agricultural soils after afforestation: a meta-analysis. *Global Change Biology, 16*(1), 439-453. doi:10.1111/j.1365-2486.2009.01930.x

[84] LeBauer, D. S., & Treseder, K. K. (2008). Nitrogen limitation of net primary productivity in terrestrial ecosystems is globally distributed. *Ecology, 89*(2), 371-379. doi:10.1890/06-2057.1

[85] Lee, M., Manning, P., Rist, J., Power, S. A., & Marsh, C. (2010). A global comparison of grassland biomass responses to CO2 and nitrogen enrichment. *Philosophical Transactions of the Royal Society B-Biological Sciences, 365*(1549), 2047-2056. doi:10.1098/rstb.2010.0028

[86] Li, H., Shen, H. H., Chen, L. Y., Liu, T. Y., Hu, H. F., Zhao, X., . . . Fang, J. Y. (2016). Effects of shrub encroachment on soil organic carbon in global grasslands. *Scientific Reports*, *6*, 9. doi:10.1038/srep28974

[87] Li, W. B., Jin, C. J., Guan, D. X., Wang, Q. K., Wang, A. Z., Yuan, F. H., & Wu, J. B. (2015). The effects of simulated nitrogen deposition on plant root traits: A meta-analysis. *Soil Biology & Biochemistry, 82*, 112-118. doi:10.1016/j.soilbio.2015.01.001

[88] Li, W. B., Wu, J. B., Bai, E., Jin, C. J., Wang, A. Z., Yuan, F. H., & Guan, D. X. (2016). Response of terrestrial carbon dynamics to snow cover change: A meta-analysis of experimental manipulation (II). *Soil Biology & Biochemistry, 103*, 388-393. doi:10.1016/j.soilbio.2016.09.017

[89] Li, Y. E., Shi, S. W., Waqas, M. A., Zhou, X. X., Li, J. L., Wan, Y. F., . . . Wilkes, A. (2018). Long-term (>= 20 years) application of fertilizers and straw return enhances soil carbon storage: a meta-analysis. *Mitigation and Adaptation Strategies for Global Change, 23*(4), 603-619. doi:10.1007/s11027-017-9751-2

[90] Liao, C. Z., Luo, Y. Q., Fang, C. M., Chen, J. K., & Li, B. (2012). The effects of plantation practice on soil properties based on the comparison between natural and planted forests: a meta-analysis. *Global Ecology and Biogeography, 21*(3), 318-327. doi:10.1111/j.1466-8238.2011.00690.x

[91] Liao, C. Z., Luo, Y. Q., Fang, C. M., & Li, B. (2010). Ecosystem Carbon Stock Influenced by Plantation Practice: Implications for Planting Forests as a Measure of Climate Change Mitigation. *Plos One, 5*(5), 6. doi:10.1371/journal.pone.0010867

[92] Liao, C. Z., Peng, R. H., Luo, Y. Q., Zhou, X. H., Wu, X. W., Fang, C. M., . . . Li, B. (2008). Altered ecosystem carbon and nitrogen cycles by plant invasion: a meta-analysis. *New Phytologist, 177*(3), 706-714. doi:10.1111/j.1469-8137.2007.02290.x

[93] Lin, D. L., Xia, J. Y., & Wan, S. Q. (2010). Climate warming and biomass accumulation of terrestrial plants: a meta-analysis. *New Phytologist, 188*(1), 187-198. doi:10.1111/j.1469-8137.2010.03347.x

[94] Liu, C., Lu, M., Cui, J., Li, B., & Fang, C. M. (2014). Effects of straw carbon input on carbon dynamics in agricultural soils: a meta-analysis. *Global Change Biology, 20*(5), 1366-1381. doi:10.1111/gcb.12517

[95] Liu, L. L., & Greaver, T. L. (2009). A review of nitrogen enrichment effects on three biogenic GHGs: the CO2 sink may be largely offset by stimulated N2O and CH4 emission. *Ecology Letters, 12*(10), 1103-1117. doi:10.1111/j.1461-0248.2009.01351.x

[96] Liu, L. L., & Greaver, T. L. (2010). A global perspective on belowground carbon dynamics under nitrogen enrichment. *Ecology Letters, 13*(7), 819-828. doi:10.1111/j.1461-0248.2010.01482.x

[97] Liu, L. L., Wang, X., Lajeunesse, M. J., Miao, G. F., Piao, S. L., Wan, S. Q., . . . Deng, M. F. (2016). A cross-biome synthesis of soil respiration and its determinants under simulated precipitation changes. *Global Change Biology, 22*(4), 1394-1405. doi:10.1111/gcb.13156

[98] Liu, S. W., Ji, C., Wang, C., Chen, J., Jin, Y. G., Zou, Z. H., . . . Zou, J. W. (2018). Climatic role of terrestrial ecosystem under elevated CO2: a bottom-up greenhouse gases budget. *Ecology Letters, 21*(7), 1108-1118. doi:10.1111/ele.13078

[99] Liu, S. W., Zhang, Y. J., Zong, Y. J., Hu, Z. Q., Wu, S., Zhou, J., . . . Zou, J. W. (2016). Response of soil carbon dioxide fluxes, soil organic carbon and microbial biomass carbon to biochar amendment: a meta-analysis. *Global Change Biology Bioenergy, 8*(2), 392-406. doi:10.1111/gcbb.12265

[100] Liu, X., Yang, T., Wang, Q., Huang, F. R., & Li, L. H. (2018). Dynamics of soil carbon and nitrogen stocks after afforestation in arid and semi-arid regions: A meta-analysis. *Science of the Total Environment, 618*, 1658-1664. doi:10.1016/j.scitotenv.2017.10.009

[101] Lu, M., Zhou, X. H., Luo, Y. Q., Yang, Y. H., Fang, C. M., Chen, J. K., & Li, B. (2011). Minor stimulation of soil carbon storage by nitrogen addition: A meta-analysis. *Agriculture Ecosystems & Environment, 140*(1-2), 234-244. doi:10.1016/j.agee.2010.12.010

[102] Lu, M., Zhou, X. H., Yang, Q., Li, H., Luo, Y. Q., Fang, C. M., . . . Li, B. (2013). Responses of ecosystem carbon cycle to experimental warming: a meta-analysis. *Ecology, 94*(3), 726-738. doi:10.1890/12-0279.1

[103] Lu, Y. H., Chen, L. D., & Fu, B. J. (2008). Land-cover effects on red soil rehabilitation in China: a meta-analysis. *Progress in Physical Geography, 32*(5), 491-502. doi:10.1177/0309133308098942

[104] Luo, G. W., Li, L., Friman, V. P., Guo, J. J., Guo, S. W., Shen, Q. R., & Ling, N. (2018). Organic amendments increase crop yields by improving microbe-mediated soil functioning of agroecosystems: A meta-analysis. *Soil Biology & Biochemistry, 124*, 105-115. doi:10.1016/j.soilbio.2018.06.002

[105] Luo, Y. Q., Hui, D. F., & Zhang, D. Q. (2006). Elevated CO2 stimulates net accumulations of carbon and nitrogen in land ecosystems: A meta-analysis. *Ecology, 87*(1), 53-63. doi:10.1890/04-1724

[106] Luo, Z. K., Wang, E. L., & Sun, O. J. (2010). Can no-tillage stimulate carbon sequestration in agricultural soils? A meta-analysis of paired experiments. *Agriculture Ecosystems & Environment, 139*(1-2), 224-231. doi:10.1016/j.agee.2010.08.006

[107] Ma, D. D., Chen, L., Qu, H. C., Wang, Y. L., Misselbrook, T., & Jiang, R. (2018). Impacts of plastic film mulching on crop yields, soil water, nitrate, and organic carbon in Northwestern China: A meta-analysis. *Agricultural Water Management, 202*, 166-173. doi:10.1016/j.agwat.2018.02.001

[108] Maia, S. M. F., Carvalho, J. L. N., Cerri, C. E. P., Lal, R., Bernoux, M., Galdos, M. V., & Cerri, C. C. (2013). Contrasting approaches for estimating soil carbon changes in Amazon and Cerrado biomes. *Soil & Tillage Research, 133*, 75-84. doi:10.1016/j.still.2013.06.002

[109] Maillard, E., & Angers, D. A. (2014). Animal manure application and soil organic carbon stocks: a meta-analysis. *Global Change Biology, 20*(2), 666-679. doi:10.1111/gcb.12438

[110] Manley, J., Van Kooten, G. C., Moeltner, K., & Johnson, D. W. (2005). Creating carbon offsets in agriculture through no-till cultivation: A meta-analysis of costs and carbon benefits. *Climatic Change, 68*(1-2), 41-65. doi:10.1007/s10584-005-6010-4

[111] Martin, P. A., Newton, A. C., & Bullock, J. M. (2017). Impacts of invasive plants on carbon pools depend on both species' traits and local climate. *Ecology, 98*(4), 1026-1035. doi:10.1002/ecy.1711/suppinfo

[112] McDaniel, M. D., Tiemann, L. K., & Grandy, A. S. (2014). Does agricultural crop diversity enhance soil microbial biomass and organic matter dynamics? A meta-analysis. *Ecological Applications, 24*(3), 560-570. doi:10.1890/13-0616.1

[113] Moreno-Mateos, D., Power, M. E., Comin, F. A., & Yockteng, R. (2012). Structural and Functional Loss in Restored Wetland Ecosystems. *Plos Biology, 10*(1), 8. doi:10.1371/journal.pbio.1001247

[114] Nave, L. E., Swanston, C. W., Mishra, U., & Nadelhoffer, K. J. (2013). Afforestation Effects on Soil Carbon Storage in the United States: A Synthesis. *Soil Science Society of America Journal, 77*(3), 1035-1047. doi:10.2136/sssaj2012.0236

[115] Nave, L. E., Vance, E. D., Swanston, C. W., & Curtis, P. S. (2009). Impacts of elevated N inputs on north temperate forest soil C storage, C/N, and net N-mineralization. *Geoderma, 153*(1-2), 231-240. doi:10.1016/j.geoderma.2009.08.012

[116] Nave, L. E., Vance, E. D., Swanston, C. W., & Curtis, P. S. (2010). Harvest impacts on soil carbon storage in temperate forests. *Forest Ecology and Management, 259*(5), 857-866. doi:10.1016/j.foreco.2009.12.009

[117] Nave, L. E., Vance, E. D., Swanston, C. W., & Curtis, P. S. (2011). Fire effects on temperate forest soil C and N storage. *Ecological Applications, 21*(4), 1189-1201. doi:10.1890/10-0660.1

[118] Nayak, D., Saetnan, E., Cheng, K., Wang, W., Koslowski, F., Cheng, Y. F., . . . Smith, P. (2015). Management opportunities to mitigate greenhouse gas emissions from Chinese agriculture. *Agriculture Ecosystems & Environment, 209*, 108-124. doi:10.1016/j.agee.2015.04.035

[119] Nie, M., Lu, M., Bell, J., Raut, S., & Pendall, E. (2013). Altered root traits due to elevated CO2: a meta-analysis. *Global Ecology and Biogeography, 22*(10), 1095-1105. doi:10.1111/geb.12062

[120] Nyawira, S. S., Nabel, J., Don, A., Brovkin, V., & Pongratz, J. (2016). Soil carbon response to land-use change: evaluation of a global vegetation model using observational meta-analyses. *Biogeosciences, 13*(19), 5661-5675. doi:10.5194/bg-13-5661-2016

[121] Obade, V. D., & Lal, R. (2014). Using meta-analyses to assess pedo-variability under different land uses and soil management in central Ohio, USA. *Geoderma, 232*, 56-68. doi:10.1016/j.geoderma.2014.04.030

[122] Ogle, S. M., Breidt, F. J., & Paustian, K. (2005). Agricultural management impacts on soil organic carbon storage under moist and dry climatic conditions of temperate and tropical regions. *Biogeochemistry, 72*(1), 87-121. doi:10.1007/s10533-004-0360-2

[123] Olsson, M. T., Erlandsson, M., Lundin, L., Nilsson, T., Nilsson, A., & Stendahl, J. (2009). Organic Carbon Stocks in Swedish Podzol Soils in Relation to Soil Hydrology and Other Site Characteristics. *Silva Fennica, 43*(2), 209-222. doi:10.14214/sf.207

[124] Peng, X., Peng, Y., Yue, K., & Deng, Y. G. (2017). Different Responses of Terrestrial C, N, and P Pools and C/N/P Ratios to P, NP, and NPK Addition: a Meta-Analysis. *Water Air and Soil Pollution, 228*(6), 13. doi:10.1007/s11270-017-3383-8

[125] Peng, Y. F., Guo, D. L., & Yang, Y. H. (2017). Global patterns of root dynamics under nitrogen enrichment. *Global Ecology and Biogeography, 26*(1), 102-114. doi:10.1111/geb.12508

[126] Peng, Y. F., Peng, Z. P., Zeng, X. T., & Houx, J. H. (2019). Effects of nitrogen-phosphorus imbalance on plant biomass production: a global perspective. *Plant and Soil, 436*(1-2), 245-252. doi:10.1007/s11104-018-03927-5

[127] Powers, J. S., Corre, M. D., Twine, T. E., & Veldkamp, E. (2011). Geographic bias of field observations of soil carbon stocks with tropical land-use changes precludes spatial extrapolation. *Proceedings of the National Academy of Sciences of the United States of America, 108*(15), 6318-6322. doi:10.1073/pnas.1016774108

[128] Qin, Z. C., Dunn, J. B., Kwon, H. Y., Mueller, S., & Wander, M. M. (2016). Soil carbon sequestration and land use change associated with biofuel

production: empirical evidence. *Global Change Biology Bioenergy, 8*(1), 66-80. doi:10.1111/gcbb.12237

[129] Ren, C. J., Chen, J., Lu, X. J., Doughty, R., Zhao, F. Z., Zhong, Z. K., . . . Ren, G. X. (2018). Responses of soil total microbial biomass and community compositions to rainfall reductions. *Soil Biology & Biochemistry, 116*, 4-10. doi:10.1016/j.soilbio.2017.09.028

[130] Ren, C. J., Zhao, F. Z., Shi, Z., Chen, J., Han, X. H., Yang, G. H., . . . Ren, G. X. (2017). Differential responses of soil microbial biomass and carbon-degrading enzyme activities to altered precipitation. *Soil Biology & Biochemistry, 115*, 1-10. doi:10.1016/j.soilbio.2017.08.002

[131] Ribeiro, A. A., Adams, C., Manfredini, S., Aguilar, R., & Neves, W. A. (2015). Dynamics of soil chemical properties in shifting cultivation systems in the tropics: a meta-analysis. *Soil Use and Management, 31*(4), 474-482. doi:10.1111/sum.12224

[132] Romero-Olivares, A. L., Allison, S. D., & Treseder, K. K. (2017). Soil microbes and their response to experimental warming over time: A meta-analysis of field studies. *Soil Biology & Biochemistry, 107*, 32-40. doi:10.1016/j.soilbio.2016.12.026

[133] Sagrilo, E., Jeffery, S., Hoffland, E., & Kuyper, T. W. (2015). Emission of CO2 from biochar-amended soils and implications for soil organic carbon. *Global Change Biology Bioenergy, 7*(6), 1294-1304. doi:10.1111/gcbb.12234

[134] Sainju, U. M. (2016). A Global Meta-Analysis on the Impact of Management Practices on Net Global Warming Potential and Greenhouse Gas Intensity from Cropland Soils. *Plos One, 11*(2), 26. doi:10.1371/journal.pone.0148527

[135] Schadel, C., Bader, M. K. F., Schuur, E. A. G., Biasi, C., Bracho, R., Capek, P., . . . Wickland, K. P. (2016). Potential carbon emissions dominated by carbon dioxide from thawed permafrost soils. *Nature Climate Change, 6*(10), 950-+. doi:10.1038/nclimate3054

[136] Schulte-Uebbing, L., & de Vries, W. (2018). Global-scale impacts of nitrogen deposition on tree carbon sequestration in tropical, temperate, and boreal forests: A meta-analysis. *Global Change Biology, 24*(2), E416-E431. doi:10.1111/gcb.13862

[137] Shi, L. L., Feng, W. T., Xu, J. C., & Kuzyakov, Y. (2018). Agroforestry systems: Meta-analysis of soil carbon stocks, sequestration processes, and future potentials. *Land Degradation & Development, 29*(11), 3886-3897. doi:10.1002/ldr.3136

[138] Shi, S. W., Peng, C. H., Wang, M., Zhu, Q. A., Yang, G., Yang, Y. Z., . . . Zhang, T. L. (2016). A global meta-analysis of changes in soil carbon, nitrogen, phosphorus and sulfur, and stoichiometric shifts after forestation. *Plant and Soil, 407*(1-2), 323-340. doi:10.1007/s11104-016-2889-y

[139] Shi, S. W., Zhang, W., Zhang, P., Yu, Y. Q., & Ding, F. (2013). A synthesis of change in deep soil organic carbon stores with afforestation of agricultural soils. *Forest Ecology and Management, 296*, 53-63. doi:10.1016/j.foreco.2013.01.026

[140] Sileshi, G. W. (2016). The magnitude and spatial extent of influence of Faidherbia albida trees on soil properties and primary productivity in drylands. *Journal of Arid Environments, 132*, 1-14. doi:10.1016/j.jaridenv.2016.03.002

[141] Sileshi, G. W., Arshad, M. A., Konate, S., & Nkunika, P. O. Y. (2010). Termite-induced heterogeneity in African savanna vegetation: mechanisms

and patterns. *Journal of Vegetation Science, 21*(5), 923-937. doi:10.1111/j.1654-1103.2010.01197.x

[142] Sillen, W. M. A., & Dieleman, W. I. J. (2012). Effects of elevated CO2 and N fertilization on plant and soil carbon pools of managed grasslands: a meta-analysis. *Biogeosciences, 9*(6), 2247-2258. doi:10.5194/bg-9-2247-2012

[143] Skinner, C., Gattinger, A., Muller, A., Mader, P., Fliessbach, A., Stolze, M., . . . Niggli, U. (2014). Greenhouse gas fluxes from agricultural soils under organic and non-organic management - A global meta-analysis. *Science of the Total Environment, 468*, 553-563. doi:10.1016/j.scitotenv.2013.08.098

[144] Song, X. Z., Peng, C. H., Jiang, H., Zhu, Q. A., & Wang, W. F. (2013). Direct and Indirect Effects of UV-B Exposure on Litter Decomposition: A Meta-Analysis. *Plos One, 8*(6), 9. doi:10.1371/journal.pone.0068858

[145] Song, X. Z., Peng, C. H., Zhou, G. M., Jiang, H., & Wang, W. F. (2014). Chinese Grain for Green Program led to highly increased soil organic carbon levels: A meta-analysis. *Scientific Reports, 4*, 7. doi:10.1038/srep04460

[146] Song, Y., Zou, Y. C., Wang, G. P., & Yu, X. F. (2017). Altered soil carbon and nitrogen cycles due to the freeze-thaw effect: A meta-analysis. *Soil Biology & Biochemistry, 109*, 35-49. doi:10.1016/j.soilbio.2017.01.020

[147] Sun, J. F., Peng, C. H., St Onge, B., Berninger, F., McCaughey, H., & Lei, X. D. (2011). META-ANALYSIS OF THE EFFECTS OF SOIL WARMING ON SOIL RESPIRATION IN FOREST ECOSYSTEMS. *Polish Journal of Ecology, 59*(4), 709-715.

[148] Swanepoel, C. M., van der Laan, M., Weepener, H. L., du Preez, C. C., & Annandale, J. G. (2016). Review and meta-analysis of organic matter in cultivated soils in southern Africa. *Nutrient Cycling in Agroecosystems, 104*(2), 107-123. doi:10.1007/s10705-016-9763-4

[149] Tang, S. M., Guo, J. X., Li, S. C., Li, J. H., Xie, S., Zhai, X. J., . . . Wang, K. (2019). Synthesis of soil carbon losses in response to conversion of grassland to agriculture land. *Soil & Tillage Research, 185*, 29-35. doi:10.1016/j.still.2018.08.011

[150] Tang, S. M., Wang, K., Xiang, Y. Z., Tian, D. S., Wang, J. S., Liu, Y. S., . . . Niu, S. L. (2019). Heavy grazing reduces grassland soil greenhouse gas fluxes: A global meta-analysis. *Science of the Total Environment, 654*, 1218-1224. doi:10.1016/j.scitotenv.2018.11.082

[151] Tang, S. M., Zhang, Y. J., Zhai, X. J., Wilkes, A., Wang, C. J., & Wang, K. (2018). Effect of grazing on methane uptake from Eurasian steppe of China. *Bmc Ecology, 18*, 7. doi:10.1186/s12898-018-0168-x

[152] Thomas, S. C., & Gale, N. (2015). Biochar and forest restoration: a review and meta-analysis of tree growth responses. *New Forests, 46*(5-6), 931-946. doi:10.1007/s11056-015-9491-7

[153] Tian, D. H., Wang, H., Sun, J., & Niu, S. L. (2016). Global evidence on nitrogen saturation of terrestrial ecosystem net primary productivity. *Environmental Research Letters, 11*(2), 9. doi:10.1088/1748-9326/11/2/024012

[154] Tian, D. S., Xiang, Y. Z., Wang, B. X., Li, M. L., Liu, Y. S., Wang, J. S., . . . Niu, S. L. (2018). Cropland abandonment enhances soil inorganic nitrogen retention and carbon stock in China: A meta-analysis. *Land Degradation & Development, 29*(11), 3898-3906. doi:10.1002/ldr.3137

[155] van der Kooi, C. J., Reich, M., Low, M., De Kok, L. J., & Tausz, M. (2016). Growth and yield stimulation under elevated CO2 and drought: A meta-analysis on crops. *Environmental and Experimental Botany, 122*, 150-157. doi:10.1016/j.envexpbot.2015.10.004

[156] van Groenigen, J. W., Lubbers, I. M., Vos, H. M. J., Brown, G. G., De Deyn, G. B., & van Groenigen, K. J. (2014). Earthworms increase plant production: a meta-analysis. *Scientific Reports, 4*, 7. doi:10.1038/srep06365

[157] Van Groenigen, J. W., Van Groenigen, K. J., Koopmans, G. F., Stokkermans, L., Vos, H. M. J., & Lubbers, I. M. (2019). How fertile are earthworm casts? A meta-analysis. *Geoderma, 338*, 525-535. doi:10.1016/j.geoderma.2018.11.001

[158] van Groenigen, K. J., Osenberg, C. W., & Hungate, B. A. (2011). Increased soil emissions of potent greenhouse gases under increased atmospheric CO2. *Nature, 475*(7355), 214-U121. doi:10.1038/nature10176

[159] van Groenigen, K. J., Osenberg, C. W., Terrer, C., Carrillo, Y., Dijkstra, F. A., Heath, J., . . . Hungate, B. A. (2017). Faster turnover of new soil carbon inputs under increased atmospheric CO2. *Global Change Biology, 23*(10), 4420-4429. doi:10.1111/gcb.13752

[160] van Groenigen, K. J., Qi, X., Osenberg, C. W., Luo, Y. Q., & Hungate, B. A. (2014). Faster Decomposition Under Increased Atmospheric CO2 Limits Soil Carbon Storage. *Science, 344*(6183), 508-509. doi:10.1126/science.1249534

[161] van Groenigen, K. J., van Kessel, C., & Hungate, B. A. (2013). Increased greenhouse-gas intensity of rice production under future atmospheric conditions. *Nature Climate Change, 3*(3), 288-291. doi:10.1038/nclimate1712

[162] Vicente-Vicente, J. L., Garcia-Ruiz, R., Francaviglia, R., Aguilera, E., & Smith, P. (2016). Soil carbon sequestration rates under Mediterranean woody crops using recommended management practices: A meta-analysis. *Agriculture Ecosystems & Environment, 235*, 204-214. doi:10.1016/j.agee.2016.10.024

[163] Virto, I., Barre, P., Burlot, A., & Chenu, C. (2012). Carbon input differences as the main factor explaining the variability in soil organic C storage in no-tilled compared to inversion tilled agrosystems. *Biogeochemistry, 108*(1-3), 17-26. doi:10.1007/s10533-011-9600-4

[164] Wan, X. H., Xiao, L. J., Vadeboncoeur, M. A., Johnson, C. E., & Huang, Z. Q. (2018). Response of mineral soil carbon storage to harvest residue retention depends on soil texture: A meta-analysis. *Forest Ecology and Management, 408*, 9-15. doi:10.1016/j.foreco.2017.10.028

[165] Wang, J., Liu, L. L., Wang, X., & Chen, Y. W. (2015). The interaction between abiotic photodegradation and microbial decomposition under ultraviolet radiation. *Global Change Biology, 21*(5), 2095-2104. doi:10.1111/gcb.12812

[166] Wang, J. Y., Xiong, Z. Q., & Kuzyakov, Y. (2016). Biochar stability in soil: meta-analysis of decomposition and priming effects. *Global Change*

*Biology Bioenergy, 8*(3), 512-523. doi:10.1111/gcbb.12266
[167] Wang, Q. K., Zhong, M. C., & Wang, S. L. (2012). A meta-analysis on the response of microbial biomass, dissolved organic matter, respiration, and N mineralization in mineral soil to fire in forest ecosystems. *Forest Ecology and Management, 271*, 91-97. doi:10.1016/j.foreco.2012.02.006
[168] Wang, X., Liu, L. L., Piao, S. L., Janssens, I. A., Tang, J. W., Liu, W. X., . . . Xu, S. (2014). Soil respiration under climate warming: differential response of heterotrophic and autotrophic respiration. *Global Change Biology, 20*(10), 3229-3237. doi:10.1111/gcb.12620
[169] Wang, X. Y., McConkey, B. G., VandenBygaart, A. J., Fan, J. L., Iwaasa, A., & Schellenberg, M. (2016). Grazing improves C and N cycling in the Northern Great Plains: a meta-analysis. *Scientific Reports, 6*, 9. doi:10.1038/srep33190
[170] Wang, X. Z. (2007). Effects of species richness and elevated carbon dioxide on biomass accumulation: a synthesis using meta-analysis. *Oecologia, 152*(4), 595-605. doi:10.1007/s00442-007-0691-5
[171] Wei, H., Xiang, Y. Z., Liu, Y., & Zhang, J. E. (2017). Effects of sod cultivation on soil nutrients in orchards across China: A meta-analysis. *Soil & Tillage Research, 169*, 16-24. doi:10.1016/j.still.2017.01.009
[172] Wu, Z. T., Dijkstra, P., Koch, G. W., Penuelas, J., & Hungate, B. A. (2011). Responses of terrestrial ecosystems to temperature and precipitation change: a meta-analysis of experimental manipulation. *Global Change Biology, 17*(2), 927-942. doi:10.1111/j.1365-2486.2010.02302.x
[173] Xia, L. L., Lam, S. K., Wolf, B., Kiese, R., Chen, D. L., & Butterbach-Bahl, K. (2018). Trade-offs between soil carbon sequestration and reactive nitrogen losses under straw return in global agroecosystems. *Global Change Biology, 24*(12), 5919-5932. doi:10.1111/gcb.14466
[174] Xia, L. L., Lam, S. K., Yan, X. Y., & Chen, D. L. (2017). How Does Recycling of Livestock Manure in Agroecosystems Affect Crop Productivity, Reactive Nitrogen Losses, and Soil Carbon Balance? *Environmental Science & Technology, 51*(13), 7450-7457. doi:10.1021/acs.est.6b06470
[175] Xiang, Y. Z., Deng, Q., Duan, H. L., & Guo, Y. (2017). Effects of biochar application on root traits: a meta-analysis. *Global Change Biology Bioenergy, 9*(10), 1563-1572. doi:10.1111/gcbb.12449
[176] Xiong, D. P., Shi, P. L., Zhang, X. Z., & Zou, C. B. (2016). Effects of grazing exclusion on carbon sequestration and plant diversity in grasslands of China A meta-analysis. *Ecological Engineering, 94*, 647-655. doi:10.1016/j.ecoleng.2016.06.124
[177] Xu, S., Liu, L. L., & Sayer, E. J. (2013). Variability of above-ground litter inputs alters soil physicochemical and biological processes: a meta-analysis of litterfall-manipulation experiments. *Biogeosciences, 10*(11), 7423-7433. doi:10.5194/bg-10-7423-2013
[178] Yan, G. Y., Mu, C. C., Xing, Y. J., & Wang, Q. G. (2018). Responses and mechanisms of soil greenhouse gas fluxes to changes in precipitation intensity and duration: a meta-analysis for a global perspective. *Canadian Journal of Soil Science, 98*(4), 591-603. doi:10.1139/cjss-2018-0002
[179] Yan, L., Zhou, G. S., & Zhang, F. (2013). Effects of Different Grazing Intensities on Grassland Production in China: A Meta-Analysis. *Plos One,*

*8*(12), 9. doi:10.1371/journal.pone.0081466

[180] Yendrek, C. R., Leisner, C. P., & Ainsworth, E. A. (2013). Chronic ozone exacerbates the reduction in photosynthesis and acceleration of senescence caused by limited N availability in Nicotiana sylvestris. *Global Change Biology, 19*(10), 3155-3166. doi:10.1111/gcb.12237

[181] Yu, L. F., Huang, Y., Sun, F. F., & Sun, W. J. (2017). A synthesis of soil carbon and nitrogen recovery after wetland restoration and creation in the United States. *Scientific Reports, 7*, 9. doi:10.1038/s41598-017-08511-y

[182] Yuan, Z. Y., Jiao, F., Shi, X. R., Sardans, J., Maestre, F. T., Delgado-Baquerizo, M., . . . Penuelas, J. (2017). Experimental and observational studies find contrasting responses of soil nutrients to climate change. *Elife, 6*, 19. doi:10.7554/eLife.23255

[183] Yue, K., Peng, C. H., Yang, W. Q., Peng, Y., Fang, J. M., & Wu, F. Z. (2015). Study type and plant litter identity modulating the response of litter decomposition to warming, elevated CO2, and elevated O-3: A meta-analysis. *Journal of Geophysical Research-Biogeosciences, 120*(3), 441-451. doi:10.1002/2014jg002885

[184] Yue, K., Peng, Y., Peng, C. H., Yang, W. Q., Peng, X., & Wu, F. Z. (2016). Stimulation of terrestrial ecosystem carbon storage by nitrogen addition: a meta-analysis. *Scientific Reports, 6*, 10. doi:10.1038/srep19895

[185] Zhang, B. C., Zhou, X. H., Zhou, L. Y., & Ju, R. T. (2015). A global synthesis of below-ground carbon responses to biotic disturbance: a meta-analysis. *Global Ecology and Biogeography, 24*(2), 126-138. doi:10.1111/geb.12235

[186] Zhang, T. A., Luo, Y. Q., Chen, H. Y. H., & Ruan, H. H. (2018). Responses of litter decomposition and nutrient release to N addition: A meta-analysis of terrestrial ecosystems. *Applied Soil Ecology, 128*, 35-42. doi:10.1016/j.apsoil.2018.04.004

[187] Zhang, W. D., Wang, X. F., & Wang, S. L. (2013). Addition of External Organic Carbon and Native Soil Organic Carbon Decomposition: A Meta-Analysis. *Plos One, 8*(2), 6. doi:10.1371/journal.pone.0054779

[188] Zhang, X. Z., Guan, D. X., Li, W. B., Sun, D., Jin, C. J., Yuan, F. H., . . . Wu, J. B. (2018). The effects of forest thinning on soil carbon stocks and dynamics: A meta-analysis. *Forest Ecology and Management, 429*, 36-43. doi:10.1016/j.foreco.2018.06.027

[189] Zhang, X. Z., Shen, Z. X., & Fu, G. (2015). A meta-analysis of the effects of experimental warming on soil carbon and nitrogen dynamics on the Tibetan Plateau. *Applied Soil Ecology, 87*, 32-38. doi:10.1016/j.apsoil.2014.11.012

[190] Zhao, H., Sun, B. F., Jiang, L., Lu, F., Wang, X. K., & Ouyang, Z. Y. (2015). How can straw incorporation management impact on soil carbon storage? A meta-analysis (vol 20, pg 1545, 2015). *Mitigation and Adaptation Strategies for Global Change, 20*(8), 1569-1569. doi:10.1007/s11027-014-9586-z

[191] Zhao, H., Sun, B. F., Lu, F., Wang, X. K., Zhuang, T., Zhang, G., & Ouyang, Z. Y. (2017). Roles of nitrogen, phosphorus, and potassium fertilizers in

carbon sequestration in a Chinese agricultural ecosystem. *Climatic Change, 142*(3-4), 587-596. doi:10.1007/s10584-017-1976-2

[192] Zhao, X., Liu, S. L., Pu, C., Zhang, X. Q., Xue, J. F., Zhang, R., . . . Chen, F. (2016). Methane and nitrous oxide emissions under no-till farming in China: a meta-analysis. *Global Change Biology, 22*(4), 1372-1384. doi:10.1111/gcb.13185

[193] Zhao, X., Pu, C., Ma, S. T., Liu, S. L., Xue, J. F., Wang, X., . . . Zhang, H. L. (2019). Management-induced greenhouse gases emission mitigation in global rice production. *Science of the Total Environment, 649*, 1299-1306. doi:10.1016/j.scitotenv.2018.08.392

[194] Zheng, F. Y., & Peng, S. L. (2001). Meta-analysis of the response of plant ecophysiological variables to doubled atmospheric CO2 concentrations. *Acta Botanica Sinica, 43*(11), 1101-1109.

[195] Zhong, Y. Q. W., Yan, W. M., & Shangguan, Z. P. (2016). The effects of nitrogen enrichment on soil CO2 fluxes depending on temperature and soil properties. *Global Ecology and Biogeography, 25*(4), 475-488. doi:10.1111/geb.12430

[196] Zhou, D., Zhao, S. Q., Liu, S., & Oeding, J. (2013). A meta-analysis on the impacts of partial cutting on forest structure and carbon storage. *Biogeosciences, 10*(6), 3691-3703. doi:10.5194/bg-10-3691-2013

[197] Zhou, G. Y., Zhou, X. H., He, Y. H., Shao, J. J., Hu, Z. H., Liu, R. Q., . . . Hosseinibai, S. (2017). Grazing intensity significantly affects belowground carbon and nitrogen cycling in grassland ecosystems: a meta-analysis. *Global Change Biology, 23*(3), 1167-1179. doi:10.1111/gcb.13431

[198] Zhou, G. Y., Zhou, X. H., Zhang, T., Du, Z. G., He, Y. H., Wang, X. H., . . . Xu, C. Y. (2017). Biochar increased soil respiration in temperate forests but had no effects in subtropical forests. *Forest Ecology and Management, 405*, 339-349. doi:10.1016/j.foreco.2017.09.038

[199] Zhou, L. Y., Zhou, X. H., Shao, J. J., Nie, Y. Y., He, Y. H., Jiang, L. L., . . . Bai, S. H. (2016). Interactive effects of global change factors on soil respiration and its components: a meta-analysis. *Global Change Biology, 22*(9), 3157-3169. doi:10.1111/gcb.13253

[200] Zhou, L. Y., Zhou, X. H., Zhang, B. C., Lu, M., Luo, Y. Q., Liu, L. L., & Li, B. (2014). Different responses of soil respiration and its components to nitrogen addition among biomes: a meta-analysis. *Global Change Biology, 20*(7), 2332-2343. doi:10.1111/gcb.12490

[201] Zhou, T., Li, L., Zhang, X., Zheng, J., Joseph, S., & Pan, G. (2016). Changes in organic carbon and nitrogen in soil with metal pollution by Cd, Cu, Pb and Zn: a meta-analysis. *European Journal of Soil Science, 67*(2), 237-246. doi:10.1111/ejss.12327

[202] Zhou, X. H., Zhou, L. Y., Nie, Y. Y., Fu, Y. L., Du, Z. G., Shao, J. J., . . . Wang, X. H. (2016). Similar responses of soil carbon storage to drought and irrigation in terrestrial ecosystems but with contrasting mechanisms: A meta-analysis. *Agriculture Ecosystems & Environment, 228*, 70-81. doi:10.1016/j.agee.2016.04.030

[203] Zhou, Z. H., Wang, C. K., & Luo, Y. Q. (2018a). Effects of forest degradation on microbial communities and soil carbon cycling: A global meta-analysis. *Global Ecology and Biogeography, 27*(1), 110-124. doi:10.1111/geb.12663

[204] Zhou, Z. H., Wang, C. K., & Luo, Y. Q. (2018b). Response of soil microbial communities to altered precipitation: A global synthesis. *Global Ecology and Biogeography, 27*(9), 1121-1136. doi:10.1111/geb.12761

[205] Zhu, L. Q., Li, J., Tao, B. R., & Hu, N. J. (2015). Effect of different fertilization modes on soil organic carbon sequestration in paddy fields in South China: A meta-analysis. *Ecological Indicators*, *53*, 144-153. doi:10.1016/j.ecolind.2015.01.038

[206] Zinn, Y. L., Lal, R., & Resck, D. V. S. (2005). Changes in soil organic carbon stocks under agriculture in Brazil. *Soil & Tillage Research, 84*(1), 28-40. doi:10.1016/j.still.2004.08.007
